# Identification of methodological issues regarding direct impact indicators of COVID-19: a rapid scoping review on morbidity, severity and mortality

**DOI:** 10.1093/eurpub/ckae072

**Published:** 2024-07-01

**Authors:** Cesar Garriga, Teresa Valero-Gaspar, Carmen Rodriguez-Blazquez, Asuncion Diaz, Péter Bezzegh, Šárka Daňková, Brigid Unim, Luigi Palmieri, Martin Thiβen, Richard Pentz, Šeila Cilović-Lagarija, Anes Jogunčić, Rodrigo Feteira-Santos, Jakov Vuković, Jane Idavain, Anda Curta, Petru Sandu, Matej Vinko, Maria João Forjaz

**Affiliations:** National Centre for Epidemiology, Carlos III Health Institute, Madrid, Spain; National Centre for Epidemiology, Carlos III Health Institute, Madrid, Spain; National Centre for Epidemiology, Carlos III Health Institute, Madrid, Spain; Neurodegenerative Diseases of the Centre for Biomedical Network Research (CIBERNED), Madrid, Spain; National Centre for Epidemiology, Carlos III Health Institute, Madrid, Spain; CIBER Thematic Area of Infectious Diseases (CIBERINFEC), Madrid, Spain; National Institute for Health Services (OKFO), Budapest, Hungary; Institute of Health Information and Statistics of the Czech Republic (“IHIS CR”), Praha, Czech Republic; Italian National Institute of Health (ISS), Rome, Italy; Italian National Institute of Health (ISS), Rome, Italy; Robert Koch Institute (RKI), Berlin, Germany; Austrian National Public Health Institute (GÖG), Vienna, Austria; Institute of Public Health of the Federation of BiH (ZZJZ FBiH), Mostaru, Bosnia and Herzegovina; Institute of Public Health of the Federation of BiH (ZZJZ FBiH), Mostaru, Bosnia and Herzegovina; Área Disciplinar Autónoma de Bioestatística, Faculdade de Medicina, Universidade de Lisboa, Lisbon, Portugal; Instituto de Saúde Ambiental, Faculdade de Medicina, Universidade de Lisboa, Lisbon, Portugal; Croatian Institute of Public Health (HZJZ), Zagreb, Croatia; National Institute for Health Development (TAI), Tallinn, Estonia; National Institute of Public Health (INSP), Bucuresti, Romania; National Institute of Public Health (INSP), Bucuresti, Romania; National Institute of Public Health (NIJZ), Ljubljana, Slovenia; National Centre for Epidemiology, Carlos III Health Institute, Madrid, Spain; Research Network on Chronicity, Primary Care and Health Promotion (RICAPPS), Madrid, Spain

## Abstract

**Background:**

During the first epidemic wave, COVID-19 surveillance focused on quantifying the magnitude and the escalation of a growing global health crisis. The scientific community first assessed risk through basic indicators, such as the number of cases or rates of new cases and deaths, and later began using other direct impact indicators to conduct more detailed analyses. We aimed at synthesizing the scientific community’s contribution to assessing the direct impact of the COVID-19 pandemic on population health through indicators reported in research papers.

**Methods:**

We conducted a rapid scoping review to identify and describe health indicators included in articles published between January 2020 and June 2021, using one strategy to search PubMed, EMBASE and WHO COVID-19 databases. Sixteen experts from European public health institutions screened papers and retrieved indicator characteristics. We also asked in an online survey how the health indicators were added to and used in policy documents in Europe.

**Results:**

After reviewing 3891 records, we selected a final sample of 67 articles and 233 indicators. We identified 52 (22.3%) morbidity indicators from 33 articles, 105 severity indicators (45.1%, 27 articles) and 68 mortality indicators (29.2%, 51). Respondents from 22 countries completed 31 questionnaires, and the majority reported morbidity indicators (29, 93.5%), followed by mortality indicators (26, 83.9%).

**Conclusions:**

The indicators collated here might be useful to assess the impact of future pandemics. Therefore, their measurement should be standardized to allow for comparisons between settings, countries and different populations.

## Introduction

The impact of the current coronavirus disease (COVID-19) global pandemic will extend for years.^1^ According to the WHO coronavirus (COVID-19) dashboard, the global death toll was 6 978 175 (accessed: 8 November 2023), with about one-third of coronavirus deaths having occurred in Europe alone.^2^ Older people and people with chronic pre-existing conditions have been reported to be at higher risk of severe COVID-19 leading to hospitalization, admission to intensive care and death. Nevertheless, the EU/EEA death rate is low compared with the pandemic maximum.^3^

The rapid worldwide response to the COVID-19 pandemic, has produced a vast amount of data from surveillance systems, health surveys and research. However, raw data is not informative enough to take public health actions. Data needs to be summarized by creating public health indicators to produce relevant information, which in turn should be interpreted to generate knowledge. This knowledge, or key messages, must be properly reported to decision-makers, who can then turn knowledge into actions.^4^

Several practical considerations for developing and choosing health indicators were taken around the world during the COVID-19 crisis, such as risk/benefit assessments considering the intensity of transmission or the health system’s capacity to respond.^5^ Currently, it is worthwhile to consider the challenges of comparing health indicators to assess the direct effects of COVID-19 on public health. For instance, testing policy differences make it difficult to compare numbers of cases between countries. Comparing the impact of COVID-19 across countries and studies involves categorizing the indicators used, based on how they are measured, their definition, purpose, unit of measurement and frequency of measurement. For instance, a wide range of different indicators were used to report severe cases requiring hospitalization. Grouping these indicators into categories would make it easier to interpret the results. It is difficult to compare indicators that are supposedly the same if they have different names, are measured and defined differently, or their purpose, unit of measurement and frequency of measurement differ from one another.

This study was part of the Population Health Information Research Infrastructure (PHIRI, https://www.phiri.eu/). PHIRI was developed to facilitate and generate the best available evidence for research on the health and wellbeing of populations impacted by COVID-19.

We aimed at providing a synthesis of the evidence assessing the direct impact of COVID-19, grouping health indicators into morbidity, severity or mortality, and identifying advantages and disadvantages of these indicators. We conducted a rapid scoping review on published articles and one online survey to investigate how health indicators were integrated and used in policy monitoring documents or decision tools.

## Methods

### Protocol and registration

A study protocol following the ‘Preferred Reporting Items for Systematic Reviews and Meta-analysis Protocols (PRISMA-P 2015) Statement’ is available at the Open Science Framework.^6^^,^^7^

### Eligibility criteria

The following Population, Concept and Context (PCC) framework was devised to inform the search strategy:

- Population: general population, patients, hospitalized patients, the dead, residents in care homes, older population.
- Concept: health indicators related to the direct impact of COVID-19 (e.g. incidence/prevalence, hospitalization, ICU admission, mortality or basic reproductive number).
- Context: representative samples of countries, regions or administrative units; multicentre studies; big data; measure of health during the pandemic; peer-reviewed articles published in English between January 2020 and June 2021.

Our review included observational studies considering cohort, case-control, cross-sectional and ecological designs. We also welcomed routinely collected health data sources (patient registries, disease registries, primary care databases, pharmacy data or cancer registries), as well as *ad hoc* research databases. An essential criterion for this rapid scoping review was choosing articles with direct impact health indicators of COVID-19 from which measurement methods could be drawn. We excluded articles reporting from one single centre, except for reference centres that received samples from others or tested their community.

**Figure 1:**
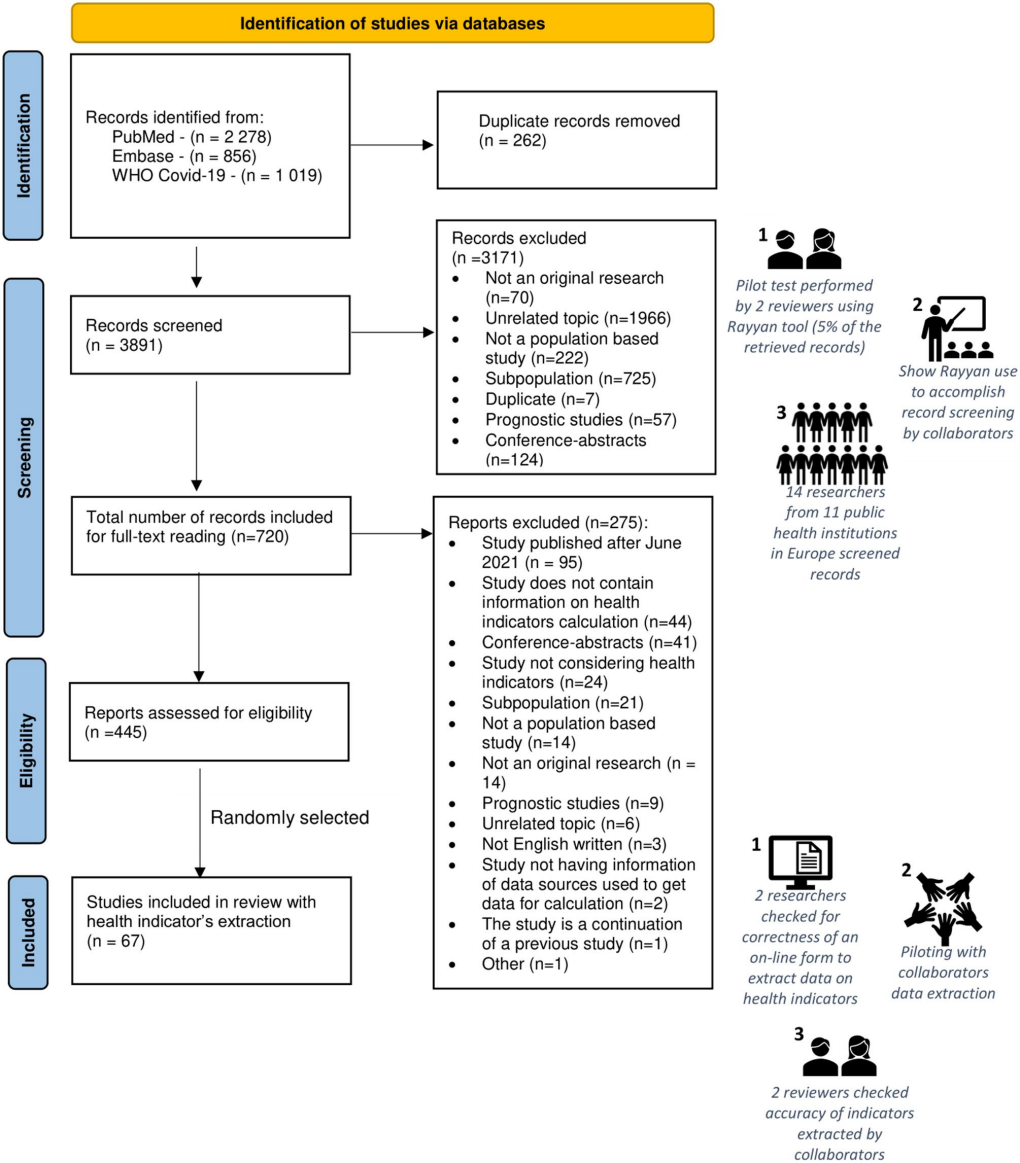
PRISMA 2020 flow diagram showing the selection process for the rapid scoping review of direct impact indicators of COVID-19

Health indicator selection involved three phases (screening, full-text reading and health-indicator extraction; figure 1) with specific exclusion criteria for each stage (Supplementary material S1).

### Information sources

PubMed and EMBASE were searched on 29 October 2021, and the WHO COVID-19 database on 2 November 2021. The PRESS statement was followed to check the appropriateness of electronic literature search strategies (Supplementary material Appendix A).^8^ The search strategies were peer-reviewed by an experienced librarian from the Spanish National Health Science Library (VJP). Search strategies were adapted for using the specific search tools available for each database. The search strategies included filters developed by expert documentalists from the United States National Library of Medicine, such as the COVID-19 filters.^9^ Search results were exported to the Rayyan systematic review management software.^10^

### Selection of sources of evidence and data charting process

#### Title/abstract screening phase

Rayyan was used for detecting and removing duplicate citations, as well as for accepting or ruling out titles and abstracts. Fourteen researchers from eleven public health institutions in Europe participated in the screening phase (figure 1). Records were rated as ‘included’, ‘excluded’ or ‘maybe’. Reviewers disagreed in 11.3% of cases, which were resolved by consensus.

#### Full-text reading phase

The articles chosen for the full-text reading phase were distributed among 12 researchers to continue the study selection process. A data charting form was developed to ask for study characteristics.^8^ Researchers used this form to add potential indicators of direct impact of COVID-19 when an article was considered appropriate for the next phase involving indicator extraction. Doubtful articles were read by peers from the group to decide about their inclusion.

#### Indicator data extraction

An online indicator-charting form was also developed. Two researchers revised accuracy of indicator extraction from five articles assigned to each collaborator. All researchers examined a random sample of approximately 15% of the articles selected during the full-text reading phase to expedite data charting. In addition, two researchers checked that all the required fields for each indicator were correctly populated. Some articles were discarded at this stage because they lacked information on indicators of direct impact of COVID-19.

### Data items

Data were drawn at study and indicator levels. Variables collected at study level included (i) article identification; (ii) geographical area; (iii) study period; (iv) study design; (v) type of sample; (vi) category of indicator identified (morbidity, severity, mortality or composite) and types of indicators within those categories. Categories were created based on the COVID-19 impact framework (Supplementary material S2). Information on whether the article reported confirmed SARS-CoV-2 diagnosis was also collected. Indicator level variables requested are listed in Supplementary material S3.

Information gathered at the study level and at the indicator level was compared for concordance. Raw data collected from the full-text reading phase and health-indicator extraction phase were linked and debugged. New variables categorizing information from the original variables were created using Stata v.17 (Stata Corporation, 2021).

### Synthesis of results

Results were presented at the study and indicator levels. Tables showed frequencies of articles and their indicators by categories for each variable. An article could have one or more indicators. Categories were ordered by number of articles for study level variables, and by number of indicators for variables collecting data about indicators. World map was plotted using the ggplot2 R-package and fed with number of articles contributing to this rapid scoping review.^11^ We grouped health indicators into morbidity, severity, mortality or a combination of the other three categories (composite). At the indicator level, results are shown stratified by these categories.

### Policy monitoring and decision tools

An online survey was developed to gather health indicators used in policy monitoring documents or decision tools and their characteristics. Collaborators from the 30 countries involved in PHIRI project were asked to complete the survey or recruit a national public health expert. Researchers were recruited from national public health institutes or health ministries (see Acknowledgement section). Experts completed one survey for each document they selected. Instructions were provided in the survey and examples given to explain the meaning of a policy document within the context of this research. Twenty-four public health experts from 22 European countries assessed the use of health indicators in their national policy monitoring documents or decision tools.

## Results

### Selection of studies and indicators

After ruling out 262 duplicate citations, we identified a total of 3891 records. We excluded 3171 records by screening titles and abstracts, leaving us 720 records for full-text reading. Of these, 275 were omitted after reading the articles in full. The remaining 445 articles matched the inclusion criteria for retrieving information regarding health indicators of direct impact. A random sample of them (67 articles, 15%) were selected for health-indicator extraction (figure 1, Supplementary material Appendixes B and C).

### Study characteristics

Of the 67 selected articles used for health-indicator extraction, 60 (89.5%) reported on 24 single countries, while 7 (10.5%) referred to many countries (e.g. in Africa). Most articles focused on USA or some of its states or cities (15; 22.4%), followed by those alluding to several countries (6; 9.0%) and those dedicated to India (5; 7.5%) (table 1). By WHO Regions, most of the studies referred to the Americas and the European region (19; 8.4% and 18; 26.9%, respectively). Almost 20% of the studies finished in June 2020, matching the first epidemic wave and lockdown (Supplementary material S4). The 57 articles reporting a study period spanned from 1 to 19 months, lasting a median of 4 months (interquartile range: 2 and 5 months). Cohort design was the most popular design implemented (33 studies; 49.2%), while the other designs considered were cross-sectional and ecological (both 17; 25.4%). Most of the study samples were drawn from general population (37; 55.2%), followed by hospitalized patients (14; 20.9%). Diagnosis of SARS-CoV-2 was confirmed by polymerase chain reaction (PCR, the gold standard test), antigen tests or chest radiographies for cases reported in 52 articles (77.6%). However, 15 articles (22.4%) did not report the type of confirmation, or mixed confirmed and non-confirmed patients. Three seroprevalence studies were selected before COVID-19 vaccines were available. Therefore, these studies indicated the estimated number of people previously infected with SARS-CoV-2.

**Table 1:** Countries and WHO-regions of studies using indicators of direct impact of COVID-19, January 2020 to June 2021

Characteristic	Number of articles/studies (*n* = 67)	%	Number of indicators (*n* = 233)	%
Study country				
USA	15	22.4	60	25.8
India	5	7.5	14	6.0
UK	4	6.0	20	8.6
China	4	6.0	13	5.6
South Korea	4	6.0	26	11.2
Italy	3	4.5	11	4.7
Iran	3	4.5	12	5.2
Denmark	2	3.0	10	4.3
France	2	3.0	3	1.3
Indonesia	2	3.0	4	1.7
Norway	2	3.0	8	3.4
Spain	2	3.0	10	4.3
Andorra	1	1.5	1	0.4
Brazil	1	1.5	3	1.3
Colombia	1	1.5	1	0.4
Iraq	1	1.5	2	0.9
Japan	1	1.5	3	1.3
Mexico	1	1.5	1	0.4
Oman	1	1.5	1	0.4
Pakistan	1	1.5	1	0.4
Peru	1	1.5	1	0.4
Philippines	1	1.5	10	4.3
Poland	1	1.5	2	0.9
Turkey	1	1.5	2	0.9
Worldwide	6	9.0	10	4.3
Africa	1	1.5	4	1.7
Study WHO region				
Americas	19	28.4	66	28.3
European region	18	26.9	67	28.8
Western Pacific Region	10	14.9	52	22.3
South-East Asia Region	7	10.5	18	7.7
Eastern Mediterranean Region	6	9.0	16	6.9
Worldwide	6	9.0	10	4.3
African region	1	1.5	4	1.7

### Indicator characteristics

We identified a total of 233 direct impact COVID-19 indicators. The number of indicators available per study varied from one to thirteen, and the median number of indicators drawn per paper was 3 (IQR: 2–4). The papers did not clearly state indicator names, and our researchers assigned names after examining the methods and results sections, as well as tables and figures and supplementary material. Subcategories of indicators grouped together diverse indicators, e.g. ‘% confirmed cases’, ‘case prevalence (cumulative reported cases/10 000 population)’ and ‘cases per 100 000 persons’ (Supplementary material Appendix C).

Most of the indicators were classified as severity indicators (105, 45.1%; from 27 articles) (table 2), mainly implemented in hospitals (95 out of 105, 85.7%). We identified 68 mortality indicators (29.2%), half of which were measured using general population samples (35 out of 68, 51.5%). Morbidity indicators (52, 22.3%) were principally drawn from the general population (41 out of 52, 78.9%).

**Table 2:** Type of indicators related to direct impact of COVID-19, January 2020 to June 2021

Characteristic	Number of articles/studies (*n*)	%^a^	Number of indicators (*n*)	%
Category of indicator				
Morbidity	33	49.3	52	22.3
Severity	27	40.3	105	45.1
Mortality	51	76.1	68	29.2
Composite	5	7.5	8	3.4
Type of morbidity indicator				
New cases in the population	15	45.5	18	34.6
Positivity rate	12	36.4	14	26.9
New and pre-existing cases divided by population	7	21.2	7	13.5
Percentage symptomatic/asymptomatic	3	9.1	4	7.7
Secondary attack rate	3	9.1	3	5.8
Growth rate	1	3.0	1	1.9
Infection case ratio	1	3.0	1	1.9
Reproductive number	1	3.0	1	1.9
Space-time cluster	1	3.0	1	1.9
Type of severity indicator				
Ventilation procedures	16	59.3	37	35.2
Mechanical ventilation	14	37.8^b^	22	59.5
Supplemental oxygen	9	24.3^b^	14	37.8
ECMO	6	16.2^b^	7	18.9
Type of ventilation procedure not reported	2	5.4^b^	3	8.1
Clinical outcomes/complications	10	37.0	15	14.3
ARDS/acute respiratory failure	5	33.3^c^	5	33.3
Acute kidney injury	3	20.0^c^	3	20.0
Pneumonia	3	20.0^c^	4	26.7
Dyspnoea	2	13.3^c^	2	13.3
Multiorgan failure	2	13.3^c^	2	13.3
Septic shock	2	13.3^c^	2	13.3
ICU	15	55.6	17	16.2
LOS at hospital	11	40.7	15	14.3
Hospitalization	10	37.0	11	10.5
Type of treatment (renal replacement, palliative care)	4	14.8	4	3.8
Length ventilation	1	3.7	1	1.0
Other severity classifications	5	18.5	5	4.8
Type of mortality indicator				
Fatality rate	36	70.6	40	58.8
Mortality rate	19	37.3	24	35.3
Time to death	2	3.9	2	2.9
Mean daily increase in deaths until the peak in mortality	1	2.0	1	1.5
YLL	1	2.0	1	1.5
Type of composite indicator				
Mortality, severity	3	60.0	4	50.0
Morbidity, mortality, severity	1	20.0	3	37.5
Morbidity, severity	1	20.0	1	12.5

ARDS: acute respiratory distress syndrome; ECMO: extracorporeal membrane oxygenation; ICU: Intensive Care Unit; LOS: length of stay; YLL: years of life lost.

a Percentage of papers having the indicators by category, e.g.: 33 papers had indicators of morbidity among a total of 67 papers, i.e. equals to 49.3% of the papers (total of percentages is higher than 100%).

b Percentages calculated over the 16 papers reporting indicators of ventilation procedures.

c Percentages calculated over the 10 papers reporting indicators of ‘clinical outcomes or complications’, non-excluding categories (total of percentages for ‘clinical outcomes or complications’ is higher than 100%).

Data from COVID-19 epidemiological surveys or registries were the most common method of feeding the indicators (89, 38.2%), mainly for morbidity indicators (26, 50.0%) and composite indicators (4, 50.0%) (Supplementary material S4). However, secondary sources were the most common when considering all of them (national registries, insurance claims, hospital or primary care records, or civil registries, i.e. 109, 46.8%). Of these, international or national registries were more frequently used when describing morbidity (16, 30.8%) and mortality (21, 30.9%) (Supplementary material S4). These indicators were often stratified by age (104; 44.6%) and sex (96; 41.2%). Stratification by socioeconomic status was mainly used for morbidity indicators (15; 30.0%) while comorbidities were used for severity indicators (30; 38.5%) (Supplementary material S4).

The most frequently reported indicator measurement strengths were: exhaustive data collection (97; 41.6%), large sample (83; 35.6%) and representativeness (73; 31.3%). Morbidity indicators presented the highest percentages on large sample and representativeness (Supplementary material S4). However, 27 articles (40.3%) did not mention the indicator’s strengths. Reported indicator measurement limitations were: missing data (68; 29.2%), mainly reported for severity indicators (47; 47.5%); lack of representativeness (63; 27.0%), mainly for severity indicators (39, 39.4%); and SARS-CoV-2 infection diagnosis not stated (43; 18.5%), mainly for mortality indicators (16; 23.5%) (Supplementary material S4). No limitations were mentioned for 32 indicators (13.7%) from 13 papers (19.4%).

### Indicators used in policy monitoring or decision tools

The respondents completed a total of 36 questionnaires, each responding to between 1 and 5 surveys. We discarded five questionnaires because they referred to indirect impact indicators, such as access to health services. Most of the selected documents were on ‘prevention and care of COVID-19 patients’ (*n* = 12, 38.7%) (Supplementary material Appendix D and Supplementary material S5). Five of the contributions were dashboards (16.1%), whereas seven were weekly reports (22.6%). Most of the identified indicators were morbidity indicators (29, 93.5%, non-excluding categories) followed by mortality indicators (26, 83.9%) (Supplementary material S5). Almost all the documents and tools reported were primary data sources (29, 93.5%). The most referenced area and period were country and week (29, 93.5% and 24, 77.4%, respectively). The indicators were mostly stratified by age (28, 90.3%), sex and geographic area (25, 80.6%, both). The most reported strength was exhaustive data collection (22, 71.0%). The contributors to this survey found few documents reporting indicator limitations (4, 12.9%). Limitations regarding missing data and diagnosis of the SARS-CoV-2 were not well defined (6, 19.4%, both limitations) (Supplementary material S5).

## Discussion

The category with the widest variety of indicators was ‘severity’, followed by the ‘mortality’ and ‘morbidity’ categories in the articles included in the scoping review (January 2020 - June 2021). The policy documents and decision tools reported in the survey mainly assessed COVID-19 impact using morbidity indicators, followed by mortality indicators (October 2022). The three most used indicators found in the rapid scoping review were two indicators of mortality, ‘fatality rate’ and ‘mortality rate;’ and one indicator of severity, ‘proportion of patients requiring mechanical ventilation.’ According to our survey, the three indicators used most often were two morbidity (‘new cases’ and ‘positivity rate’); and one mortality (‘mortality rate’) indicators.

### Morbidity indicators

Morbidity indicators aim to estimate the occurrence of diseases, lesions and impairment in populations. Incidence is employed for acute illnesses of short duration which are curable or end in death.^4^ The ‘rate of new confirmed cases nationwide per 100 000 persons’ indicator was used to measure the incidence of notified COVID-19 cases in the community by the ECDC surveillance system.^12^ Both the ECDC and the WHO dashboards estimated COVID-19 morbidity as incidence values per 100 000 population over the past 14 days.^2^^,^^13^ However, most studies identified in the scoping review estimated ‘cumulative incidence’ for study periods ranging from 3 to 10 months when reporting new cases in the population, instead of 14-day periods.^1^^,^^14–19^ Most of the studies used convenience periods (‘cumulative incidences’ in a defined period), instead of reporting daily, weekly or monthly incidence.^20–22^ We found a different approach in a study analysing the whole of Africa, that measured the ‘weekly growth rate’ between one week compared with the previous week.^22^ The ECDC also used this reporting method to evaluate weekly changes in the epidemic wave.^3^ Policy monitoring documents and decision tools also used new cases. Overall, national weekly reports and dashboards included these indicators. We classified three seroepidemiological studies in the ‘morbidity’ category to ascertain positivity for antibodies and prevalence. They differed from other prevalence studies in that they included asymptomatic cases or incomplete ascertainment of patients with symptoms.^23^ Hence, seroepidemiological studies provided information on the proportion of the population that remained susceptible.

### Severity indicators

Considering that ‘admission to hospital’ might be a proxy for disease severity, the rate of hospitalized COVID-19 cases was used as an indicator of the disease burden in the population.^12^ Severity was mainly surveyed by rates of hospital admissions’ and ‘ICU admissions’ per 100 000 people weekly by the ECDC.^3^ Hospital admission rates were employed as a proxy for primary care quality because high admission rates may indicate low care coordination or low care continuity. They may also point to structural constraints such as the insufficient number of general practitioners.^24^ The CDC used the indicator ‘new COVID-19 admissions per 100 000 population (7-day total)’ and, when available, ‘percent of emergency department visits due to COVID-19’ based on the syndromic surveillance.^25^

### Mortality indicators

Mortality is a key indicator of severity and a measure of effectiveness of control measures for COVID-19.^12^ ‘Case fatality rate’ estimates the severity of a disease, but only if the estimation of cases is reliable.^26^ The articles included measured ‘fatality rate’ using symptomatic cases or positive tests.^14^^,^^27–29^ Therefore, the measurement based on symptomatic cases did not include cases with mild or no symptoms, and COVID-19 case fatality was overestimated. Another indicator of mortality, ‘mortality rate’, was obtained using population denominators varying between 10 000 and 1 000 000 habitants.^1^^,^^14^^,^^15^^,^^18^^,^^28^^,^^30–33^ In line with these mortality indicators, the WHO coronavirus (COVID-19) dashboard also measured mortality rate per 100 000 people.^2^ Both mortality rate and fatality rate were indicators described by public health institutions and governments across Europe, as shown by the survey on policy monitoring documents and decision tools.

### Strengths and limitations of the literature review, policy monitoring documents and decision tools

The main strength of this study is that it obtained results from scientific articles and documents describing or disseminating policy monitoring and decision tools. The scientific papers reviewed have provided a large number of indicators, covering all main categories of indicators and collecting, if not all, then at least the vast majority of indicators assessing the direct impact of the COVID-19 crisis. The online survey aimed to learn how policy documents included and used the indicators. Practical lessons can be drawn from the policy documents.

At hospital level, in-house databases, and serological surveys (primary data sources) stood out in the scientific studies. This was reflected in a higher collection of severity indicators (e.g. percentage of ventilation devices), information that was readily available in these settings. These indicators might not have been available to policymakers during the acute phase of the epidemic from their regular data sources, until new surveillance systems could be in place. Therefore, results published in peer-reviewed scientific journals could provide wider information than information collected through passive national public health surveillance and other national statistics to better inform policymaking.

The vastly different indicator names and definitions hindered their categorization, consequently limiting data comparison. In addition, not all indicators extracted from scientific papers evaluating COVID-19 direct impact could be suitable for assessing other emerging infectious diseases. For example, the mechanical ventilation requirement was mainly relevant in the context of a respiratory infection such as COVID-19. Despite the substantial number of European researchers involved in selecting of sources of evidence, certain difficulties arose in harmonizing raw data across several tasks, including time to collect, organize, debug and synthesize a large number of indicators and characteristics. For this reason, we agreed with our collaborators to select a random sample of papers (*n* = 67) to conduct the rapid scoping review, instead of extracting information from all studies included in the full-text reading phase. We used simple random sampling to avoid bias in small samples and to produce more representative results. Another limitation was the variation in the epidemic status by country.^26^ Publications also included variation by circulating strain and epidemiological situation.^34^ The selected indicators were strongly dependent on contextual factors affecting both its values and their interpretation, e.g. changing criteria for hospital admission.

Based on the difficulties found to identify and retrieve indicators and their characteristics, we recommend the following for scientific publications: give clear identification of the health indicators; set focus on indicators widely used in surveillance and research; point out if an indicator is currently used to monitor epidemics by surveillance systems; highlight variables of stratification (e.g. ethnicity) to guide public health measures; justify the use of new indicators by stating which gap they are covering; provide a thorough definition of the indicator and its characteristics in the methods section; and provide alternative names if the indicator is introduced for the first time and there is no current consensus to name it.

### Conclusions and implications for public health

We have obtained a wide variability of indicators reporting morbidity, severity or mortality. Using morbidity, severity and mortality subcategories could facilitate better identification of appropriate indicators, depending on the type of study to be conducted. Our research has highlighted the need for researchers to agree on a list of indicators to include in their studies, so that results can be compared across studies and countries for specific future crises. This list of indicators should be adjusted, depending on how easy it is to get precise information in diverse nations with varying degrees of development in their health information systems. Researchers contributing with publications that use harmonized indicators could speed up findings beyond individual investigations, to generate aggregated and cross-national information for decision-makers in future health crises. Shortlists of indicators such as the European Core Health Indicators (ECHI) could be improved with new sets of indicators for future health crises. Moreover, scientific journals and funding bodies could support the selection of indicators from an internationally agreed shortlist when a health crisis like COVID-19 begins. This way, researchers could evaluate the vast number of technical documents and scientific publications quantitatively and cross-nationally.

## Supplementary Material

ckae072_Supplementary_Data

## Data Availability

The data underlying this article are available in the article and in its online supplementary material. Key points Indicators of direct impact of COVID-19 included in the scientific literature match those found in policy monitoring documents and decision tools, and the ones used by surveillance systems or enlisted by international health organizations to allow cross-national comparisons. Most of the indicators can be measured in many ways, and therefore can be applied to specific study needs and local particularities. However, they should be harmonized to make rapid comparisons in future pandemics or health crises. Data sources are similar, and therefore new or enhanced indicators could be added to common indicator lists such as the ECHI shortlist.
