# Supplementary material for: Identification of methodological issues regarding direct impact indicators of COVID-19: a rapid scoping review on morbidity, severity and mortality
Source: Eur J Public Health. 2024 Jul 1;34(Suppl 1):i3–i10. doi: 10.1093/eurpub/ckae072 (PMC11215319; doi:10.1093/eurpub/ckae072)
Supplement: ckae072_Supplementary_Data [file ckae072_supplementary_data.zip › ejph-2023-06-phis-0310-File007.pdf]

Supplementary material S3. Online indicator-charting form.

Description of indicators

1. Email *

2. Reference: First Author (Surname, Name)

3. Reference: Country affiliation for the First Author

4. Name of indicator

Type of indicator

Indicate why it is important to use the proposed indicator

5. Type of indicator

Mark only one oval.

- ◯ *1.* Morbidity *Go to question 6*
- ◯ *2.* Mortality *Go to question 7*
- ◯ *3.* Severity *Go to question 8*
- ◯ *4.* Composite *Go to question 11*

Morbidity

6. Morbidity

Mark only one oval.

- ◯ Incidence *Go to question 16*
- ◯ Prevalence *Go to question 14*
- ◯ Reproductive number *Go to question 12*
- ◯ Underreported infections *Go to question 12*
- ◯ Other: ____________ *Go to question 12*

Mortality

7. Mortality

Mark only one oval.

- ◯ Mortality rate (denominator= cases + non-cases) *Go to question 18*
- ◯ Fatality rate (denominator= COVID-19 cases) *Go to question 20*
- ◯ Time to death *Go to question 12*
- ◯ Other: ____________ *Go to question 14*

Severity

(Health indicators related to hospital admission and stay)

8. Severity

Mark only one oval.

- ◯ Length of stay *Go to question 12*
- ◯ Hospitalisation *Go to question 22*
- ◯ Admission to ICU *Go to question 24*
- ◯ Ventilation procedures *Go to question 9*
- ◯ Clinical outcomes/ Complications (e.g. nephropathy, stroke) *Go to question 10*
- ◯ Other: ______________________

Ventilation procedures

9. Treatment

Check all that apply.

- ☐ Mechanical ventilation
- ☐ Supplemental oxygen
- ☐ Extracorporeal membrane oxygenation (ECMO)
- ☐ Other: ______________________ *Go to question 12*

Clinical outcomes/ Complications

As a consequence of SARS-CoV-2 infection (DO NOT INCLUDE BASELINECHARACTERISTICS)

10. Clinical outcomes

Check all that apply.

- [ ] Acute respiratory distress syndrome (ARDS)/acute respiratory failure
- [ ] Hyperglycaemia
- [ ] Diabetes
- [ ] Acute kidney injury/failure
- [ ] Nephropathy
- [ ] Septic shock
- [ ] Hospital acquired pneumonia
- [ ] Anaemia
- [ ] Liver dysfunction
- [ ] Myocardial infarction
- [ ] Atrial fibrillation
- [ ] Congestive heart failure
- [ ] Disseminated intravascular coagulopathy (DIC)
- [ ] Deep venous thrombosis (DVT)
- [ ] Pulmonary thrombo-embolism (PTE)
- [ ] Cerebro vascular disorders (any)
- [ ] Stroke
- [ ] Meningitis
- [ ] Transient ischaemic attack (TIA)
- [ ] Ischaemic stroke
- [ ] Ischaemic heart disease
- [ ] Neurological disorders (excluding vascular)
- [ ] Psychiatric disorders (any)
- [ ] Organic mental disorders
- [ ] Other: ______________________

Go to question 12

Composite

Please tick 2 of them at least (e.g. Severity+Mortality)

11. Composite

Check all that apply.

- [ ] Morbidity
- [ ] Mortality
- [ ] Severity

Go to question 12

Calculation method (I)

Describe how the indicator is calculated, (could include) the numerator and the denominator, when relevant

12. Numerator

____
____
____
____
____

13. Denominator

____
____
____
____
____

Go to question 26

Prevalence calculation

Describe how the indicator is calculated: write the numerator and select the denominator from the list

14. Numerator

___

___

___

15. Denominator

Mark only one oval.

- ◯ Divided by symptomatic residents
- ◯ Divided by exposed residents
- ◯ Divided by subjects tested with RT-PCR
- ◯ Divided by subjects tested for antibodies against SARS-CoV-2
- ◯ Divided by subjects tested for IgG antibodies against SARS-CoV-2
- ◯ Divided by subjects tested for IgG antibodies against SARS-CoV-2 AND presenting symptoms suggestive of COVID-19
- ◯ Divided by subjects tested (any test)
- ◯ Divided by subjects with SARS-CoV-2
- ◯ Divided by patients diagnosed with COVID-19
- ◯ Divided by seropositive
- ◯ Other: ___

___

___

Go to question 26

Incidence calculation

Describe how the indicator is calculated: write the numerator and select the denominator from the list.

16. Numerator

17. Denominator

Mark only one oval.

- Divided by negatives in the first survey (baseline test) in a serological study
- Divided by population/ inhabitants
- Divided by insured people
- Divided by person-weeks (COVID-19 cases confirmed by laboratory tests)
- Other: ______________

Go to question 26

Mortality rate calculation

Describe how the indicator is calculated: write the numerator and select the denominator from the list.

Alternative names to mortality rate indicator:
Cumulative deaths, Cumulative mortality, In-hospital mortality, Median deaths per day, COVID-19 specific mortality...

18. Numerator

____
____
____

19. Denominator

Mark only one oval.

- ◯ Divided by population/ inhabitants
- ◯ Divided by patient/days
- ◯ Divided by person/days
- ◯ Deaths per day
- ◯ Divided by insured
- ◯ Divided by residents (nursing homes)
- ◯ Other: ______________________

Go to question 26

Fatality rate calculation

Describe how the indicator is calculated: write the numerator and select the denominator from the list.

Alternative names for fatality rate indicator:
Case-fatality rate, Proportion non-survivors in 30 days, Infection fatality risk, Infection fatality ratio, Infection fatality rate, mortality rate (absolute risk) (relative risk), case mortality rate, In-hospital mortality, Proportion non-survivors, Age standardised mortalityratio, Proportion non-survivors (ICU mortality)...

20. Numerator

Mark only one oval.

- Confirmed COVID-19 deaths
- COVID-19 deaths (any, confirmed or not confirmed)
- Excess all cause deaths
- Daily deaths
- Other: ____________________

21. Denominator

Mark only one oval.

- Divided by residents with SARS-CoV-2
- Divided by subjects positive to IgG antibodies against SARS-CoV-2
- Divided by subjects with SARS-CoV-2
- Divided by patients diagnosed with COVID-19
- Daily cases
- Divided by hospital admissions for COVID-19
- Divided by COVID-19 patients admitted to ICU
- Divided by COVID-19 patients in ICU with MECHANICAL
- VENTILATIONDivided by NON-VENTILATED COVID-19 patients in
- ICU
- Divided by COVID-19 patients requiring ICU (FiO2 ≥0.80)
- Divided by COVID-19 patients requiring High Flow oxygen (FiO2 ≥0.36)

◯ Other: ____________________

Go to question 26

Hospital admission rate calculation

Describe how the indicator is calculated: write the numerator and select the denominator from the list.

Alternative names to Hospital admission rate indicator:
Cumulative incidence of SARSCoV-2 confirmed cases admitted to hospital, rate hospital admission, burden of hospital admissions, Proportion for self-report hospitalization due to COVID-19, Proportion of patients hospitalised with COVID-19 lineage B.1.1.7, Proportion of patients hospitalised, Proportion of patients hospitalised (excluding those at ICU), Proportion of patients hospitalised for over 24 hours

22. Numerator

____

____

____

23. Denominator

Mark only one oval.

◯ Divided by cases + non-cases
◯ Divided by inhabitants
◯ Divided by random sample of inhabitants
◯ Divided by diagnosed COVID-19 cases
◯ Divided among those with whole SARS-CoV-2 genome sequenced in the population
◯ Divided by definite, suspected, and probable cases of coronavirus
◯ Divided by individuals with SARS-CoV-2 antibodies
◯ Other: ____________________

Go to question 26

ICU admission rate calculation

Describe how the indicator is calculated: write the numerator and select the denominator from the list.

24. Numerator

____

____

____

____

____

25. Denominator

Mark only one oval.

- ⬭ Divided by cases + non-cases
- ⬭ Divided by inhabitants
- ⬭ Divided by hospitalised COVID-19 cases
- ⬭ Divided by individuals with SARS-CoV-2 antibodies
- ⬭ Other: ____________________

Go to question 26

Calculation Method (II)

Describe denominator and calculation method (please write the formula as this example: patients requiring oxygen therapy due to COVID-19 during the follow-up period/Total number of people admitted to hospital with COVID-19 during the follow-up period*100)

26. 10^n (how coefficient is expressed)

Mark only one oval.

- ◯ 100 (i.e. %)
- ◯ 1000 (i.e. ‰)
- ◯ 10,000
- ◯ 100,000
- ◯ 1,000,000
- ◯ N/A (e.g. time-dependant reproduction ratio)
- ◯ Other: ______________________

27. Calculation method

____

____

____

____

____

Go to question 28

Type of indicator

See document: https://bit.ly/3tDT4xC (pages 18 and 19)

Count: gives the number of occurrence of the events being studied, within a specified time and specified place.

Ratio: shows a relationship between two numbers. It is calculated by dividing one quantity by another, which may or may not be of the same type. Types of ratios:

- Proportion: This is when the numerator in a subset of the denominator. Usually expressed as a percentage.

- Rate: The numerator is the absolute number of occurrences of the event being studied in a specified time. The denominator is the reference population (or population being studied) at the same time.

- Odds: The numerator is the proportion of the event of interest, and the denominator is the proportion of the non-event. The numerator and denominator are thus complementary proportions (p/1-p)

28. Type of indicator

Mark only one oval.

- Count
- Proportion
- Rate
- Odds
- Other: ______________________

Area of reference

Indicate the country or geographic area to which the indicator refers

29. Area of reference

Check all that apply.

- [ ] Global
- [ ] National/Country
- [ ] Region/ county/ department
- [ ] City/ municipality
- [ ] District
- [ ] Hospital
- [ ] Other: ____________________

Reference period

Specify the time period, or point in time, to which the indicator refers

30. Reference period

Mark only one oval.

- ( ) Year
- ( ) Month
- ( ) Week
- ( ) Day
- ( ) Other: ____________________

Disaggregation dimensions

The breakdown of observations within a common branch of a hierarchy to a more detailed level to that at which
detailed observations are taken. The characteristics by which data is to be disaggregated: sex, age group, ethnic origin, geographic area (country, state, province, urban/rural)...

31. Disaggregation

Check all that apply.

- [ ] Age
- [ ] Sex
- [ ] Ethnicity
- [ ] Education
- [ ] Socio economic status (SES)
- [ ] Deprivation index
- [ ] Body Mass Index (BMI)
- [ ] Comorbidities Smoking
- [ ] Travelled abroad
- [ ] Nursing home
- [ ] Geographic area (country, state, province, urban/rural...)
- [ ] Other: ____________________

Data source

Include which is the data source where numerator and denominator were obtained for doing the calculation (national registry, national census, WHO database, seroepidemiological survey, Department of Health...)

32. Numerator

____

____

33. Denominator

____

Indicator strengths

Specify the positive points in the indicator’s measurement, use, and interpretation.

Look into the discussion section of the paper and select those strengths related to the indicator.
DO NOT INCLUDE strenghts of the study if they are not associated with the health indicator.

34. Indicator strengths

Check all that apply.

- [ ] Data collection
- [ ] Large sample
- [ ] Representativeness
- [ ] Detection of asymptomatic or subclinical infection
- [ ] Random sample
- [ ] Not mentioned
- [ ] Other: ______________________

Indicator limitations

Specify the challenges or constraints in the indicator's measurement, use, and interpretation.

Look at the discussion section of the paper and select the strengths related to the indicator.
DO NOT INCLUDE strenghts of the study if they are not associated with the health indicator.

35. Indicator limitations

Check all that apply.

- [ ] Data collection bias
- [ ] Small sample
- [ ] Lack of representativeness
- [ ] Missing data
- [ ] Convenience sample
- [ ] Enrolment (participation) bias
- [ ] False-positive cases
- [ ] Not mentioned
- [ ] Lack of more granular (detailed) data (e.g. disaggregated data by region)
- [ ] Other: ____________________

Technical notes

Include all relevant matters related to the construction of the indicator than could hinder its use and interpretation (different from indicator limitations)

36. Technical notes

____

____

____
