# Supplementary material for: Identification of methodological issues regarding direct impact indicators of COVID-19: a rapid scoping review on morbidity, severity and mortality
Source: Eur J Public Health. 2024 Jul 1;34(Suppl 1):i3–i10. doi: 10.1093/eurpub/ckae072 (PMC11215319; doi:10.1093/eurpub/ckae072)
Supplement: ckae072_Supplementary_Data [file ckae072_supplementary_data.zip › ejph-2023-06-phis-0310-File008.pdf]

Supplementary material S4. Tables reporting on indicators related to direct impact of COVID-19 from papers selected in a scoping review, January 2020-June 2021.

Table S4.1. Characteristics of studies using indicators of direct impact of COVID-19, January 2020-June 2021.

Characteristic	Number of articles/studies (n=67)	%	Number of indicators (n=233)	%
**Study end (month)**				
Jan-20	1	1.5	1	0.4
Feb-20	2	3.0	6	2.6
Mar-20	4	6.0	16	6.9
Apr-20	10	14.9	47	20.2
May-20	14	20.9	47	20.2
Jun-20	14	20.9	45	19.3
Jul-20	6	9.0	28	12.0
Ago-20	3	4.5	13	5.6
Sep-20	4	6.0	6	2.6
Nov-20	2	3.0	4	1.7
Dic-20	5	7.5	14	6.0
Jan-21	1	1.5	4	1.7
Feb-21	1	1.5	2	0.9
**Study design**				
Cohort study	33	49.2	164	70.4
Cross-sectional study	17	25.4	32	13.7
Ecological study	17	25.4	37	15.9
**Study sample**				
General population	38	56.7	97	41.6

Hospitalised patients	14	20.9	78	33.5
Inpatients/Outpatients	10	14.9	38	16.3
Patients in/requiring ICU	2	3.0	14	6.0
People living in LTCF	2	3.0	5	2.2
Close contacts	1	1.5	1	0.4
**SARS-CoV-2 infection diagnosis**				
Confirmed	52	77.6	190	81.6
Not confirmed, unclear or mixed	15	22.4	43	18.5

ICU: intensive care unit; LTCF: long-term care facility

Table S4.2. Sources providing data, type of numerator and denominator on indicators of direct impact of COVID-19, January 2020-June 2021.

	Morbidity		Mortality		Severity		Composite	
	n	%	n	%	n	%	n	%
**Data source**								
COVID-19 epidemiological survey/registry	26	50.0	27	39.7	32	30.5	4	50.0
International/national/regional registry	16	30.8	21	30.9	13	12.4	1	12.5
In-house databases and serological survey	6	11.5	4	5.9	25	23.8	0	0.0
Insurance claims	1	1.9	2	2.9	15	14.3	2	25.0
Other data sources	3	5.8	14	20.6	20	19.1	1	12.5
**Denominator data source**								
COVID-19 epidemiological survey/registry	17	34.7	15	23.1	27	32.9	1	33.3
International/national/regional registry	10	20.4	20	30.8	13	15.9	2	66.7
Census	15	30.6	12	18.5	2	2.4	0	0.0
Hospital admission records	1	2.0	13	20.0	11	13.4	0	0.0
Other denominator data sources	6	12.2	5	7.7	29	35.4	0	0.0
Without denominator	3	–	3	–	23	–	5	–
**Numerator**								
Confirmed COVID-19 Deaths	0	0.0	37	54.4	9	8.6	0	0.0
Ventilation therapies (or requesting)	0	0.0	0	0.0	36	34.3	3	37.5
Confirmed cases	28	53.9	0	0.0	10	9.5	0	0.0
Hospitalised or severe	0	0.0	0	0.0	28	26.7	1	12.5
Other numerators	24	46.2	31	45.6	22	21.0	4	50.0
**Denominator** [a]								
Symptomatic cases	1	1.9	20	29.4	30	28.6	0	0.0
Positive tests	7	13.5	17	25.0	23	21.9	1	12.5
Population	24	46.2	19	27.9	3	2.9	1	12.5
Tested PCR/confirmed cases	2	3.9	2	2.9	11	10.5	1	12.5
Other denominators	15	30.6	7	10.8	15	18.3	0	0.0
Without denominator	3	–	3	–	23	–	5	–

PCR: polymerase chain reaction. [a] 34 indicators had not a denominator; percentages were calculated over the 205 indicators with a denominator.

Table S4.3. Contextual variables, strengths and limitations of indicators related to direct impact of COVID-19, January 2020-June 2021.

	Morbidity		Mortality		Severity		Composite	
	n	%	n	%	n	%	n	%
**Area of reference [a]**								
Global	2	3.9	3	4.4	0	0.0	0	0.0
National/country	11	21.2	38	55.9	66	62.9	6	75.0
Region/county/department	25	48.1	18	26.5	23	21.9	1	12.5
City/municipality	21	40.4	8	11.8	15	14.3	0	0.0
District	1	1.9	0	0.0	0	0.0	0	0.0
Hospital	1	1.9	5	7.4	6	5.7	0	0.0
Long-term care facilities	3	5.8	1	1.5	6	5.7	1	12.5
**Reference period**								
Cumulative for the period	31	59.6	47	69.1	82	78.1	8	100.0
Year	0	0.0	1	1.5	1	1.0	0	0.0
Month	12	23.1	12	17.7	21	20.0	0	0.0
Week	5	9.6	0	0.0	0	0.0	0	0.0
Day	4	7.7	6	8.8	1	1.0	0	0.0
Hour [b]	0	0.0	2	2.9	0	0.0	0	0.0
**Stratification by: [a]**								
Age	27	54.0	32	52.5	40	51.3	5	62.5
Sex	24	48.0	25	41.0	42	53.9	5	62.5
Comorbidities	8	16.0	17	27.9	30	38.5	2	25.0
Geographic area	20	40.0	24	39.3	8	10.3	0	0.0
Ethnicity	10	20.0	15	24.6	20	25.6	1	12.5
Socio economic status	15	30.0	10	16.4	6	7.7	0	0.0

**Strengths** [a]								
Exhaustive data collection	13	25.0	25	39.1	53	52.5	6	75.0
Large sample	24	46.2	21	32.8	34	33.7	4	50.0
Representativeness	21	40.4	21	32.8	28	27.7	3	37.5
Detection of asymptomatic	13	25.0	2	3.1	1	1.0	0	0.0
**Limitations** [a]								
Missing data	8	16.7	13	20.0	47	47.5	0	0.0
Lack of representativeness	10	20.8	13	20.0	39	39.4	1	12.5
SARS-CoV-2 infection Dx not clear	9	17.3	16	23.5	13	12.4	5	62.5
Enrolment (participation) bias	2	4.2	5	7.7	34	34.3	0	0.0
Data collection bias	6	12.5	12	18.5	10	10.1	3	37.5
Lack of more granular data	13	27.1	10	15.4	7	7.1	0	0.0
Small sample	0	0.0	4	6.2	25	25.3	1	12.5

Dx: diagnosis; [a] Non-excluding category (total of percentages is higher than 100%). [b] Richardson et al. estimated mortality within 48- and 72-hours.
