# Supplementary material for: Identification of methodological issues regarding direct impact indicators of COVID-19: a rapid scoping review on morbidity, severity and mortality
Source: Eur J Public Health. 2024 Jul 1;34(Suppl 1):i3–i10. doi: 10.1093/eurpub/ckae072 (PMC11215319; doi:10.1093/eurpub/ckae072)
Supplement: ckae072_Supplementary_Data [file ckae072_supplementary_data.zip › ejph-2023-06-phis-0310-File009.pdf]

Supplementary material S5. Tables of indicators related to direct impact of COVID-19 retrieved from policy monitoring and decision tool documents (October 2022).

Table S5.1. Aim of the documents.

Aim of the document	Number of documents (n)	%
**Promotion**	1	3.2
**Prevention**	7	22.6
**Care of COVID-19 patients**	1	3.2
**Promotion and prevention**	2	6.5
**Prevention and care**	12	38.7
**Promotion, prevention and care**	8	25.8

Table S5.2. Type of indicators.

Type of indicators	Number of documents (n)	%
**Type of indicators**		
Morbidity	29	93.5
Severity	20	64.5
Mortality	26	83.9
Composite	10	32.3
**Morbidity indicators**[a]		
New cases	25	86.2
Positivity rate	22	75.9
New and previous cases	17	58.6
**Severity indicators**[b]		
ICU admission	18	90.0
Length of stay	6	30.0
Ventilation procedures	6	30.0
Clinical outcomes/complications	2	10.0
Hospitalisation	2	10.0
**Mortality indicators**[c]		
Mortality rate	25	96.2
Fatality rate	13	50.0
**Items making composite indicators**[d]		
ICU admission	2	20.0
New cases	1	10.0
Positivity rate	1	10.0
New and previous cases	1	10.0
Ventilation procedures	1	10.0
Mortality rate	1	10.0

[a] Divided by the 29 documents with morbidity indicators. [b] Divided by the 20 documents with severity indicators. [c] Divided by 26 documents with mortality indicators. [d] To estimate percentage: Numerator includes number of documents where only composite indicator was reported, denominator has the 10 documents with composite indicators.

Table S5.3. Characteristics of indicators.

Type of indicators	Number of documents (n)	%
**How the indicators are mathematically expressed**		
Proportion	20	64.5
Rate	24	77.4
Count	24	77.4
**Data sources**		
Primary data source	29	93.5
Secondary data source	21	67.7
**How coefficients are expressed**		
100 (i.e. %)	28	90.3
1000 (i.e ‰)	1	3.2
10,000	1	3.2
100,000	13	41.9
1,000,000	2	6.5
N/A	3	9.7
**Area of reference**	13	50.0
National/Country	29	93.5
Region/county/department	23	74.2
City/ municipality	8	25.8
**Reference period**	2	18.2
Defined period (e.g. March 2020 to June 2020)	18	58.1
Month	5	16.1
Week	24	77.4
Day	11	35.5
**Stratification by**		
Age	28	90.3
Sex	25	80.6

Geographic area (country, state, province, urban/rural...)	25	80.6
Comorbidities	6	19.4
Socio economic status	3	9.7
Ethnicity	0	0.0
**Indicators' strengths**		
Exhaustive data collection	22	71.0
Large sample	19	61.3
Representativeness	18	58.1
Not described	4	12.9
**Indicators' limitations**		
Missing data	6	19.4
SARS-CoV-2 infection diagnosis not clear	6	19.4
Lack of representativeness	3	9.7
Enrolment (participation) bias	2	6.5
Not described	20	64.5

N/A: not applicable.
