# Supplementary material for: Identification of methodological issues regarding direct impact indicators of COVID-19: a rapid scoping review on morbidity, severity and mortality
Source: Eur J Public Health. 2024 Jul 1;34(Suppl 1):i3–i10. doi: 10.1093/eurpub/ckae072 (PMC11215319; doi:10.1093/eurpub/ckae072)
Supplement: ckae072_Supplementary_Data [file ckae072_supplementary_data.zip › ejph-2023-06-phis-0310-File010.pdf]

Appendix A. Search strategies.

PubMed Search Strategy

Date of search: 29.10.2021

Search	*Most Recent Queries*	*results*	*comment*
#1	("COVID-19" OR "COVID-19"[MeSH Terms] OR "COVID-19 Vaccines" OR "COVID-19 Vaccines"[MeSH Terms] OR "COVID-19 serotherapy" OR "COVID-19 serotherapy"[Supplementary Concept] OR "COVID-19 Nucleic Acid Testing" OR "covid-19 nucleic acid testing"[MeSH Terms] OR "COVID-19 Serological Testing" OR "covid-19 serological testing"[MeSH Terms] OR "COVID-19 Testing" OR "covid-19 testing"[MeSH Terms] OR "SARS-CoV-2" OR "sars-cov-2"[MeSH Terms] OR "Severe Acute Respiratory Syndrome Coronavirus 2" OR "NCOV" OR "2019 NCOV" OR (("coronavirus"[MeSH Terms] OR "coronavirus" OR "COV") AND 2019/11/01[PDAT] : 3000/12/31[PDAT])) *AND ((2020/01/01:2021/06/30[Date - Publication] AND "english"[Language]) NOT ("animals"[MeSH Terms] NOT "humans"[MeSH Terms]))*	*152,526 29.10.2021*	*Disease definition*<br><br>1. COVID-19 filter obtained from the National Library of Medicine. Section article filters. Limit retrieval to citations about the 2019 novel coronavirus. Category General. Filter name “LitCGeneral” = LitCGeneral[Filter] https://pubmed.ncbi.nlm.nih.gov/help<br><br>2. *Filter modified for retrieving human studies: Instead of human filter: NOT ("animals"[MeSH Terms] NOT "humans"[MeSH Terms]). Following the advice of the librarian from the Medical Library of the University Hospital of Getafe, Madrid, Spain https://bibliogetafe.com/*
#2	Epidemiologic Studies[Mesh]	*2,805,991 29.10.2021*	*Study definition – MeSH Terms*<br><br>*MeSH Terms included in Epidemiologic Studies[Mesh]*<br><br>1. Cohort studies[MeSH Terms] includes “incidence” in its entry terms:

			• Incidence Studies<br>• Incidence Study<br>• Studies, Incidence<br>• Study, Incidence<br><br>2. Cross-sectional studies[MeSH Terms] includes “prevalence” in its entry terms:<br><br>• Prevalence Studies<br>• Prevalence Study<br>• Studies, Prevalence<br>• Study, Prevalence<br><br>3. Epidemiologic Studies[MeSH Terms]<br><br>• Case-Control Studies<br>• Retrospective Studies<br>• Cohort Studies<br>• Follow-Up Studies<br>• Longitudinal Studies +<br>• Prospective Studies<br>• Retrospective Studies<br>• Controlled Before-After Studies<br>• Cross-Sectional Studies<br>• Historically Controlled Study<br>• Interrupted Time Series Analysis<br>• Seroepidemiologic Studies<br>o HIV Seroprevalence
#3	(Epidemiological Studies[tiab] OR Epidemiological Study[tiab] OR Epidemiologic Study[tiab] OR "cross-sectional"[tiab] OR "cross sectional"[tiab] OR Disease Frequency Survey*[tiab] OR "case-control"[tiab] OR "case control"[tiab] OR Case-Comp*[tiab] OR Case Comp*[tiab] OR Case-	*2,703,792*<br>*29.10.2021*	Study definition – [terms title/abstract/keywords]

	Refer*[tiab] OR Case Refer*[tiab] OR Case-Base[tiab] OR Case Base[tiab] OR cohort[tiab] OR Concurrent Stud*[tiab] OR longitudinal[tiab] OR follow?up[tiab] OR "prospective study"[tiab] OR "prospective studies"[tiab] OR "prospective design"[tiab] OR "prospective designs"[tiab] OR "retrospective study"[tiab] OR "retrospective studies"[tiab] OR "retrospective design"[tiab] OR "retrospective designs"[tiab] OR "prospective observational study"[tiab] OR "prospective observational studies"[tiab] OR "retrospective observational study"[tiab] OR "retrospective observational studies"[tiab] OR seroepidemiolog*[tiab] OR sero-epidemiolog*[tiab] OR serogroup epidemiology[tiab] OR serologic epidemiology[tiab] OR serological epidemiology[tiab] OR serotype epidemiology[tiab] OR serotypic epidemiology[tiab] OR ecological study[tiab] OR "ecological studies"[tiab] OR "ecological design"[tiab] OR "ecological designs"[tiab] OR observational[tiab] OR "observational study"[tiab] OR "observational study"[pt] OR "observational studies"[tiab] OR "observational design"[tiab] OR "observational designs"[tiab])		
#4	(#2) OR (#3)	4,027,321 *29.10.2021*	*Study definition – [MeSH Terms] + [terms title/abstract/keywords]*
#5	"Morbidity"[Mesh]	606,487 29.10.2021	*MeSH Terms included in Morbidity"[Mesh]*

			1. Morbidity includes:<br>• Basic Reproduction Number[Mesh]<br>• Incidence[Mesh]<br>• Prevalence[Mesh]
#6	R0[tiab] OR Basic reproductive number[tiab] OR time-varying reproduction number[tiab] OR reproduction number[tiab] OR prevalence*[tiab] OR incidence*[tiab] OR morbidit*[tiab] OR Attack Rate*[tiab] OR Secondary Attack[tiab] OR Person-time Rate*[tiab] OR Person time Rate*[tiab]	1,827,897 29.10.2021	*Indicator: Morbidity [terms title/abstract/keywords]*
#7	(#5) OR (#6)	2,004,075 29.10.2021	*Indicator: Morbidity [MeSH Terms] + [terms title/abstract/keywords]*
#8	"Mortality"[Mesh]	408,873 29.10.2021	*Indicator: Mortality [MeSH Terms]*
#9	mortalit*[tiab] OR case-fatality rate[tiab] OR case fatality rate[tiab] OR death rate*[tiab] OR crude death[tiab] OR excess deaths [tiab] OR excess all cause deaths [tiab] OR excess number of deaths [tiab] OR excess COVID-19-related deaths [tiab] OR infection fatality r*[tiab] OR number covid-19 deaths [tiab] OR confirmed covid-19 deaths [tiab] OR deaths hospitalization ratio[tiab] OR survival[tiab] OR death toll[tiab] OR fatal outcome[tiab]	1,785,354 29.10.2021	*Indicator: Mortality [terms title/abstract/keywords]*
#10	(#8) OR (#9)	1,936,352 29.10.2021	*Indicator: Mortality [MeSH Terms] + [terms title/abstract/keywords]*
#11	("Patient Admission"[Mesh] OR "Intensive Care Units"[Mesh] OR "Respiration, Artificial"[Mesh] OR	1,432,901 29.10.2021	*Indicator: Severity – [MeSH Terms]*

	"Inpatients"[Mesh] OR "Risk Adjustment"[Mesh] OR "Outcome Assessment, Health Care"[Mesh])		
#12	acute respiratory infection[tiab] OR Patient Admi*[tiab] OR Voluntary Admission*[tiab] OR hospital Admi*[tiab] OR intensive care[tiab] OR Respiratory Care Unit*[tiab] OR Recovery room*[tiab] OR close attention unit[tiab] OR critical care unit[tiab] OR intensive therapy unit[tiab] OR intensive treatment unit[tiab] OR special care unit[tiab] OR Respiration, Artificial[tiab] OR Artificial Respirat*[tiab] OR Ventilation, Mechanical[tiab] OR mechanical ventilation*[tiab] OR Interactive Ventilatory[tiab] OR Ventilatory Support[tiab] OR Ventilatory Assist[tiab] OR Assist Ventilation[tiab] OR Ventilation, Proportional Assist[tiab] OR invasive ventilation[tiab] OR controlled respiration[tiab] OR controlled ventilation[tiab] OR mechanical respiration[tiab] OR mechanical ventilation[tiab] OR Inpatient*[tiab] OR in-patient*[tiab] OR Hospitalised patient*[tiab] OR hospitalized patient*[tiab] OR in-hospital patient*[tiab] OR Risk Adjustment*[tiab] OR risk analysis[tiab] OR risk evaluation[tiab] OR safety assessment[tiab] OR Case-Mix Adjustment*[tiab] OR	2,782,928<br>29.10.2021	*Indicator: Severity [terms title/abstract/keywords]*

	Case Mix Adjustment*[tiab] OR Outcomes Assessment*[tiab] OR Outcome Assessment*[tiab] OR Outcomes Research[tiab] OR Outcome Stud*[tiab] OR Outcome Measure*[tiab] OR severity[tiab] OR case-hospitalization ratio[tiab] OR proportion of hospitalization[tiab]		
#13	(#11) OR (#12)	3,695,874 29.10.2021	*Indicator: Severity [MeSH Terms] + [terms title/abstract/keywords]*
#14	"Population Health"[Mesh]	39,980 *29.10.2021*	*General population definition [MeSH Terms]*
#15	(Population Health[tiab] OR populational health[tiab] OR general population[tiab] OR population stud*[tiab] OR population cohort study[tiab] OR population-based[tiab] OR community-based[tiab] OR community dwelling[tiab] OR Nationwide[tiab] OR national cohort[tiab] OR million people[tiab] OR "federal state*"[tiab] OR "state level"[tiab] OR *region**[tiab] OR countr*[tiab] OR county[tiab] OR counties[tiab] OR nation*[tiab])	3,052,622 *29.10.2021*	*General population definition [terms title/abstract/keywords]*
#16	(#14) OR (#15)	3,080,050 29.10.2021	*General population definition [MeSH Terms] + [terms title/abstract/keywords]*
#17	"Nursing Homes"[Mesh] OR Homes for the Aged [Mesh]	47,142 29.10.2021	*Nursing homes definition [MeSH Terms]*
#18	Nursing Home*[tiab] OR convalescence home*[tiab] OR convalescence hospital*[tiab] OR extended care facilit*[tiab] OR long term care facilit*[tiab] OR skilled nursing facilit*[tiab] OR Homes for the Aged[tiab] OR Geriatric facilit*[tiab] OR Old Age Home*[tiab] OR long-	57,616 29.10.2021	*Nursing homes definition [terms title/abstract/keywords]*

	term care[tiab] OR aged care home*[tiab] OR aged care facilit*[tiab] OR continuing care retirement center*[tiab] OR geriatric homes*[tiab] OR home? for the elderly[tiab] OR homes for the aged[tiab] OR housing for the elderly[tiab] OR old people home*[tiab] OR retirement center*[tiab] OR retirement centre*[tiab] OR retirement home*[tiab] OR senior residence facility*[tiab]		
#19	(#17) OR (#18)	76,487<br>29.10.2021	*Nursing homes definition [MeSH Terms] + [terms title/abstract/keywords]*
#20	((clinical[Title/Abstract] AND trial[Title/Abstract]) OR clinical trials as topic[MeSH Terms] OR clinical trial[Publication Type] OR random*[Title/Abstract] OR random allocation[MeSH Terms] OR therapeutic use[MeSH Subheading])	*5,878,395*<br>*29.10.2021*	*RCT definition*<br><br>1. RCT filter obtained from the National Library of Medicine. Optimized for sensitive/broad; sensitive/specific 99%/70%. https://pubmed.ncbi.nlm.nih.gov/help/#publication-types. Search filters based on the work of Haynes RB et al. doi: 10.1136/bmj.38068.557998
#21	("case report*"[tiab] OR ("case reports"[Publication Type] OR "case reports"[tiab]) OR "report a case"[tiab] OR ("report*"[tiab] AND ("ambulatory care facilities"[MeSH Terms] OR ("ambulatory"[tiab] AND "care"[tiab] AND "facilities"[tiab]) OR "ambulatory care facilities"[tiab] OR "clinic"[tiab] OR "clinic s"[tiab] OR "clinical"[tiab] OR "clinically"[tiab] OR "clinicals"[tiab] OR "clinics"[tiab] OR "patient*"[tiab])) OR "reported case"[tiab] OR "clinical presentation*"[tiab] OR "patient management"[tiab] OR "infected patient*"[tiab])	3,734,074<br>29.10.2021	*Case report definition*<br><br>1. Filter obtained from the National Library of Medicine. Section article filters. Filter name: LitCCaseReport. https://pubmed.ncbi.nlm.nih.gov/help/#publication-types<br>2. *Filter modified: [All Fields] changed to [tiab]*

#22	"Mental Health"[Mesh] OR "Depression"[Mesh] OR "Depressive Disorder"[Mesh] OR "Anxiety"[Mesh] OR "Anxiety Disorders"[Mesh]	393,102<br>29.10.2021	*Mental health definition [MeSH terms]*
#23	mental health[tiab] OR prevalence of depression[Title/Abstract] OR ″Psychosocial Impact″[tiab] OR ″Psychological Outcomes”[tiab] OR "Depressi*"[tiab] OR Melancholia*[tiab] OR "Anxiety"[tiab] OR Angst[tiab] OR Nervousness[tiab] OR Hypervigilance[tiab] OR Anxiousness[tiab] OR Anxieties[tiab]	683,353<br>29.10.2021	*Mental Health definition [terms title/abstract/keywords]*
#24	(#22) OR (#23)	790,677<br>29.10.2021	*Mental Health definition [MeSH Terms] + [terms title/abstract/keywords]*
#25	(#7) OR (#10) OR (#13)	6,063,073<br>29.10.2021	*Joining Indicators*
#26	(#1) AND (#4) AND ((#16) OR (#19)) AND (#25)	4,655<br>29.09.2021	*Disease + Type of study + Type of population (#16 OR #19) + Indicators*
#27	(#26) NOT preprint[pt]	4,556<br>*29*.10.2021	*Preprint definition*<br>1. Preprint filter obtained from the National Library of Medicine. https://pubmed.ncbi.nlm.nih.gov/help/#publication-types<br>*Exclusion criteria: preprints*
#28	(#27) NOT (#20)	3667<br>29.10.2021	*Exclusion criteria: RCT*
#29	(#28) NOT (#21)	2638<br>29.10.2021	*Exclusion criteria: case reports*
#30	(#29) NOT (#24)	2278<br>*29.10.2021*	*Exclusion criteria: mental health prevalence*

Steps applied for the EMBASE Search Strategy

Search interface: embase.com

Date of search: 29.10.2021

Search	Most Recent Queries	results	comment
#1	('coronavirus disease 2019'/exp OR 'covid 19' OR 'Coronavirus infection'/exp OR 'Coronavirinae'/exp OR 'Severe acute respiratory syndrome coronavirus 2'/exp OR '2019nCoV' OR 'corona virus*' OR 'coronavirus*' OR 'coronovirus*' OR '2019-nCoV' OR 'cov 2' OR '2019-nCoV' OR 'cov2' OR 'nCov 2019' OR 'nCoV' OR 'covid 19' OR 'COVID19' OR 'SARS-CoV-2' OR 'SARS2' OR ((19:ab,ti OR 2019:ab,ti OR '2019 ncov' OR beijing OR china OR 'covid 19' OR epidem*:ab,ti OR epidemic* OR (epidemies OR epidemy) OR new:ab,ti OR novel:ab,ti OR pandem* OR 'sars-cov-2' OR shanghai OR wuhan) AND ('Coronavirus infection'/exp OR 'Coronavirinae'/exp OR coronavirus* OR 'corona virus*' OR cov:ab,ti OR 'pneumonia virus*':ab,ti))) AND ([1-1-2020]/sd NOT [1-7-2021]/sd AND [english]/lim AND [embase]/lim NOT ([embase]/lim AND [medline]/lim) AND ([humans]/lim NOT [animals]/lim))	34,722 29.10.2021	Disease definition<br><br>1. Search used from https://doi.org/10.1111/acem.14048 Data supplement s1 appendix in acem14048-sup-0001-datasuppls1.pdf<br>2. Ruled out Medline papers to avoid duplicates already retrieved in the PubMed search.<br>3. Replaced: “AND [humans]/lim” by “AND ([animals]/lim NOT [humans]/lim)” for human studies based on Higgins JPT, Green S (editors). Cochrane Handbook for Systematic Reviews of Interventions Version 5.1.0 [updated March 2011]. The Cochrane Collaboration, 2011. Available from http://training.cochrane.org/handbook, Chapter 2, section 6.4. cited in https://bibliogetafe.com/2018/04/23/filtro-de-busqueda-para-estudios-en-humanos/
#2	'cross-sectional study'/exp OR 'case control study'/exp OR 'cohort analysis'/exp OR 'retrospective study'/exp	4,181,113 29.10.2021	Study definition [Emtree Terms]

	OR 'follow up'/exp OR 'longitudinal study'/exp OR 'prospective study'/exp OR 'seroepidemiology'/exp OR 'observational study'/exp		
#3	'epidemiological stud*':ti,ab,kw OR 'epidemiologic stud*':ti,ab,kw OR 'cross?sectional':ti,ab,kw OR 'disease frequency survey':ti,ab,kw OR 'case?control':ti,ab,kw OR 'control study':ti,ab,kw OR 'case?comp*':ti,ab,kw OR 'case?refer*':ti,ab,kw OR 'case?base':ti,ab,kw OR 'cohort':ti,ab,kw OR 'concurrent stud*':ti,ab,kw OR 'retrospective stud*':ti,ab,kw OR 'retrospective design*':ti,ab,kw OR 'follow?up':ti,ab,kw OR 'longitudinal':ti,ab,kw OR 'prospective stud*':ti,ab,kw OR 'prospective design*':ti,ab,kw OR 'prospective method*':ti,ab,kw OR 'seroepidemiolog*':ti,ab,kw OR 'sero-epidemiolog*':ti,ab,kw OR 'serogroup epidemiology':ti,ab,kw OR 'serologic epidemiology':ti,ab,kw OR 'serological epidemiology':ti,ab,kw OR 'serotype epidemiology':ti,ab,kw OR 'serotypic epidemiology':ti,ab,kw OR 'ecological study':ti,ab,kw OR 'ecological studies':ti,ab,kw OR 'ecological design':ti,ab,kw OR 'ecological	*3,861,148*<br>*29.10.2021*	*Study definition – [terms title/abstract/keywords]*

	designs':ti,ab,kw OR observational:ti,ab,kw		
#4	#2 OR #3	5,373,407 *29.10.2021*	*Study definition [Emtree Terms] + [terms title/abstract/keywords]*
#5	'morbidity'/exp	*402,372 29.10.2021*	*Indicator: Morbidity [Emtree Terms]*
#6	'r0':ti,ab,kw OR 'basic reproductive number':ti,ab,kw OR 'time-varying reproduction number':ti,ab,kw OR 'reproduction number':ti,ab,kw OR 'prevalence*':ti,ab,kw OR 'incidence*':ti,ab,kw OR 'morbidit*':ti,ab,kw OR 'attack rate*':ti,ab,kw OR 'secondary attack':ti,ab,kw OR 'person-time rate*':ti,ab,kw OR 'person time rate*':ti,ab,kw	*2,720,490 29.10.2021*	*Indicator: Morbidity [terms title/abstract/keywords]*
#7	#5 OR #6	*2,804,713 29.10.2021*	*Indicator: Morbidity [Emtree Terms] + [terms title/abstract/keywords]*
#8	'mortality'/exp	*1,254,233 29.10.2021*	*Indicator: Mortality [Emtree Terms]*
#9	‘mortalit*’:ti,ab,kw OR ‘case-fatality rate’:ti,ab,kw OR ‘case fatality rate’:ti,ab,kw OR ‘death rate*’:ti,ab,kw OR ‘crude death’:ti,ab,kw OR ‘excess deaths’:ti,ab,kw OR ‘excess all cause deaths’:ti,ab,kw OR ‘excess number of deaths’:ti,ab,kw OR ‘excess COVID-19-related deaths’:ti,ab,kw OR ‘infection fatality r*’:ti,ab,kw OR ‘number covid-19 deaths’:ti,ab,kw OR ‘confirmed covid-19 deaths’:ti,ab,kw OR ‘deaths hospitalization	*2,676,744 29.10.2021*	*Indicator: Mortality [terms title/abstract/keywords]*

	ratio’:ti,ab,kw OR ‘survival’:ti,ab,kw OR ‘death toll’:ti,ab,kw OR ‘fatal outcome’:ti,ab,kw		
#10	#8 OR #9	3,007,982 *29.10.2021*	*Indicator: Mortality [Emtree Terms] + [terms title/abstract/keywords]*
#11	'hospital admission'/exp OR 'intensive care unit'/exp OR 'artificial ventilation'/exp OR 'hospital patient'/exp OR 'risk assessment'/exp OR 'outcome assessment'/exp	1,949,505 29.10.2021	*Indicator: Severity [Emtree Terms]*
#12	‘acute respiratory infection’:ti,ab,kw OR ‘Patient Admi*’:ti,ab,kw OR ‘Voluntary Admission*’:ti,ab,kw OR ‘hospital Admi*’:ti,ab,kw OR ‘intensive care’:ti,ab,kw OR ‘Respiratory Care Unit*’:ti,ab,kw OR ‘Recovery room*’:ti,ab,kw OR ‘close attention unit’:ti,ab,kw OR ‘critical care unit’:ti,ab,kw OR ‘intensive therapy unit’:ti,ab,kw OR ‘intensive treatment unit’:ti,ab,kw OR ‘special care unit’:ti,ab,kw OR ‘Respiration, Artificial’:ti,ab,kw OR ‘Artificial Respirat*’:ti,ab,kw OR ‘Ventilation, Mechanical’:ti,ab,kw OR ‘mechanical ventilation*’:ti,ab,kw OR ‘Interactive Ventilatory’:ti,ab,kw OR ‘Ventilatory Support’:ti,ab,kw OR ‘Ventilatory Assist’:ti,ab,kw OR ‘Assist Ventilation’:ti,ab,kw OR ‘Ventilation, Proportional Assist’:ti,ab,kw OR ‘invasive	4,283,391 29.10.2021	*Indicator: Severity [terms title/abstract/keywords]*

	ventilation’:ti,ab,kw OR ‘controlled respiration’:ti,ab,kw OR ‘controlled ventilation’:ti,ab,kw OR ‘mechanical respiration’:ti,ab,kw OR ‘mechanical ventilation’:ti,ab,kw OR ‘Inpatient*’:ti,ab,kw OR ‘in-patient*’:ti,ab,kw OR ‘Hospitalised patient*’:ti,ab,kw OR ‘hospitalized patient*’:ti,ab,kw OR ‘in-hospital patient*’:ti,ab,kw OR ‘Risk Adjustment*’:ti,ab,kw OR ‘risk analysis’:ti,ab,kw OR ‘risk evaluation’:ti,ab,kw OR ‘safety assessment’:ti,ab,kw OR ‘Case-Mix Adjustment*’:ti,ab,kw OR ‘Case Mix Adjustment*’:ti,ab,kw OR ‘Outcomes Assessment*’:ti,ab,kw OR ‘Outcome Assessment*’:ti,ab,kw OR ‘Outcomes Research’:ti,ab,kw OR ‘Outcome Stud*’:ti,ab,kw OR ‘Outcome Measure*’:ti,ab,kw OR ‘severity’:ti,ab,kw OR ‘case-hospitalization ratio’:ti,ab,kw OR ‘proportion of hospitalization’:ti,ab,kw		
#13	#11 OR #12	5,385,924 29.10.2021	*Indicator: Severity [Emtree Terms] + [terms title/abstract/keywords]*
#14	*'population health'/exp*	4,048 29.10.2021	*General population definition [Emtree Terms]*
#15	‘Population Health’:ti,ab,kw OR ‘populational	3,972,922 29.10.2021	*General population definition [terms title/abstract/keywords]*

	health’:ti,ab,kw OR ‘general population’:ti,ab,kw OR ‘population stud*’:ti,ab,kw OR ‘population cohort study’:ti,ab,kw OR ‘population-based’:ti,ab,kw OR ‘community-based’:ti,ab,kw OR ‘community dwelling’:ti,ab,kw OR ‘Nationwide’:ti,ab,kw OR ‘national cohort’:ti,ab,kw OR ‘million people’:ti,ab,kw OR ‘federal state*‘:ti,ab,kw OR ‘state level‘:ti,ab,kw OR ‘*region**’:ti,ab,kw OR ‘countr*’:ti,ab,kw OR ‘county’:ti,ab,kw OR ‘counties’:ti,ab,kw OR ‘nation*’:ti,ab,kw		
#16	*#14 OR #15*	3,973,519 29.10.2021	*General population definition [Emtree Terms] + [terms title/abstract/keywords]*
#17	'nursing home'/exp OR 'home for the aged'/exp	65,045 29.10.2021	*Nursing homes definition [Emtree Terms]*
#18	‘Nursing Home*’:ti,ab,kw OR ‘convalescence home*’:ti,ab,kw OR ‘convalescence hospital*’:ti,ab,kw OR ‘extended care facilit*’:ti,ab,kw OR ‘long term care facilit*’:ti,ab,kw OR ‘skilled nursing facilit*’:ti,ab,kw OR ‘Homes for the Aged’:ti,ab,kw OR ‘Geriatric facilit*’:ti,ab,kw OR ‘Old Age Home*’:ti,ab,kw OR ‘long-term care’:ti,ab,kw OR ‘aged care home*’:ti,ab,kw OR ‘aged care facilit*’:ti,ab,kw OR ‘continuing care retirement center*’:ti,ab,kw OR ‘geriatric homes*’:ti,ab,kw OR ‘home? for the elderly’:ti,ab,kw OR ‘homes	76,049 29.10.2021	*Nursing homes definition [terms title/abstract/keywords]*

	for the aged':ti,ab,kw OR 'housing for the elderly':ti,ab,kw OR 'old people home*':ti,ab,kw OR 'retirement center*':ti,ab,kw OR 'retirement centre*':ti,ab,kw OR 'retirement home*':ti,ab,kw OR 'senior residence facility*':ti,ab,kw		
#19	#17 OR #18	*100,778 29.10.2021*	*Nursing homes definition [Emtree Terms] + [terms title/abstract/keywords]*
#20	('clinical':ab,ti,kw AND 'trial':ab,ti,kw) OR 'clinical trial (topic)'/exp OR 'clinical trial':it OR 'random*':ab,ti,kw OR 'randomization'/exp OR 'therapeutic use'/exp	2,209,897 *29.10.2021*	*RCT definition*<br><br>1. Based on RCT filter obtained from the National Library of Medicine. Optimized for sensitive/broad; sensitive/specific 99%/70%. https://pubmed.ncbi.nlm.nih.gov/help/#publication-types. Search filters based on the work of Haynes RB et al. doi: 10.1136/bmj.38068.557998
#21	*('case report*' OR ('case reports':it OR 'case reports') OR 'report a case' OR ('report*' AND ('outpatient department'/exp OR ('ambulatory' AND 'care' AND 'facilities') OR 'ambulatory care facilities' OR 'clinic' OR 'clinic s' OR 'clinical' OR 'clinically' OR 'clinicals' OR 'clinics' OR 'patient*')) OR 'reported case' OR 'clinical presentation*' OR 'patient management' OR 'infected patient*')*	5,839,019 *29.10.2021*	*Case report definition*<br><br>1. Filter obtained from the National Library of Medicine. Section article filters. Based on LitCCaseReport filter https://pubmed.ncbi.nlm.nih.gov/help/#publication-types
#22	'mental health'/exp OR 'depression'/exp OR 'anxiety'/exp OR 'anxiety disorder'/exp	1,001,057 29.10.2021	*Mental health definition [Emtree terms]*
#23	'mental health':ti,ab,kw OR 'prevalence of depression':ti,ab,kw OR 'Psychosocial Impact':ti,ab,kw OR	946,706 29.10.2021	*Mental Health definition [terms title/abstract/keywords]*

	‘Psychological Outcomes’:ti,ab,kw OR ‘Depressi*’:ti,ab,kw OR ‘Melancholia*’:ti,ab,kw OR ‘Anxiety’:ti,ab,kw OR ‘Angst’:ti,ab,kw OR ‘Nervousness’:ti,ab,kw OR ‘Hypervigilance’:ti,ab,kw OR ‘Anxiousness’:ti,ab,kw OR ‘Anxieties’:ti,ab,kw		
#24	#22 OR #23	1,324,453<br>29.10.2021	*Mental Health definition [Emtree Terms] + [terms title/abstract/keywords]*
#25	#7 OR #10 OR #13	8,736,136<br>29.10.2021	*Joining Indicators*
#26	#1 AND #4 AND (#16 OR #19) AND #25	1,855<br>*29.10.2021*	*Disease + Type of study + Type of population (#16 or #19) + Indicators*
#27	#26 NOT #20	1684<br>29.10.2021	*Exclusion criteria: RCT*
#28	#27 NOT #21	956<br>29.10.2021	*Exclusion criteria: case reports*
#29	#28 NOT #24	856<br>*29.10.2021*	*Exclusion criteria: mental health prevalence*

WHO COVID-19 Global literature on coronavirus disease strategy
Search interface: search.bvsalud.org

Date of search: 02.11.2021

Full Search using WHO COVID-19 Global literature on coronavirus disease:

((tw:("Epidemiological Studies")) OR (tw:("Epidemiological Study")) OR (tw:("Epidemiologic Study")) OR (tw:("cross-sectional")) OR (tw:("cross sectional")) OR (tw:("Disease Frequency" survey*)) OR (tw:("case-control")) OR (tw:("case control")) OR (tw:(case-comp*)) OR (tw:(case comp*)) OR (tw:(case-refer*)) OR (tw:(case refer*)) OR (tw:("Case-Base")) OR (tw:("Case Base")) OR (tw:(cohort)) OR (tw:(concurrent stud*)) OR (tw:(longitudinal)) OR (tw:("follow up")) OR (tw:(follow-up)) OR (tw:(prospective stud*)) OR (tw:(prospective design*)) OR (tw:(retrospective stud*)) OR (tw:(retrospective design*)) OR (tw:(seroepidemiolog*)) OR (tw:(sero-epidemiolog*)) OR (tw:("serogroup epidemiology")) OR (tw:("serologic epidemiology")) OR (tw:("serological epidemiology")) OR (tw:("serotype epidemiology")) OR (tw:("serotypic epidemiology")) OR (tw:(ecological stud*)) OR (tw:(ecological design*)) OR (tw:(observational)) OR (type_of_study:("observational_studies")) OR (tw:(incidence stud*)) OR (tw:(prevalence stud*)) OR (tw:("Controlled Before-After" stud*)) OR (tw:("Interrupted Time Series"))) AND ((tw:("Population Health")) OR (tw:("populational health")) OR (tw:("general population")) OR (tw:(population stud*)) OR (tw:("population cohort study")) OR (tw:("population-based")) OR (tw:("community-based")) OR (tw:("community dwelling")) OR (tw:(Nationwide)) OR (tw:("national cohort")) OR (tw:("million people")) OR (tw:(federal state*)) OR (tw:("state level")) OR (tw:(region*)) OR (tw:(countr*)) OR (tw:(county)) OR (tw:(counties)) OR (tw:(nation*)) OR (tw:(Nursing Home*)) OR (tw:(convalescence home*)) OR (tw:(convalescence hospital*)) OR (tw:("extended care" facilit*)) OR (tw:("long term care" facilit*)) OR (tw:("skilled nursing" facilit*)) OR (tw:("Homes for the Aged")) OR (tw:(Geriatric facilit*)) OR (tw:("Old Age" Home*)) OR (tw:("long-term care")) OR (tw:("aged care" home*)) OR (tw:("aged care" facilit*)) OR (tw:("continuing care retirement" center*)) OR (tw:(geriatric homes*)) OR (tw:(home? "for the elderly")) OR (tw:("homes for the aged")) OR (tw:("housing for the elderly")) OR (tw:("old people" home*)) OR (tw:(retirement center*)) OR (tw:(retirement centre*)) OR (tw:(retirement home*)) OR (tw:("senior residence" facility*))) AND ((tw:(R0)) OR (tw:("Basic reproductive number")) OR (tw:(reproduction number)) OR (tw:(prevalence*)) OR (tw:(incidence*)) OR (tw:(morbidit*)) OR (tw:(attack rate*)) OR (tw:("Secondary Attack")) OR (tw:("Person-time" Rate*)) OR (tw:("Person time" Rate*)) OR (tw:(mortalit*)) OR (tw:("case-fatality" rate*)) OR (tw:("case fatality" rate*)) OR (tw:(death rate*)) OR (tw:("crude death")) OR (tw:("excess deaths")) OR (tw:("excess all cause deaths")) OR (tw:("excess number of deaths")) OR (tw:("excess COVID-19-related deaths")) OR (tw:("infection fatality" r*)) OR (tw:("number covid-19 deaths")) OR (tw:("confirmed covid-19 deaths")) OR (tw:("deaths hospitalization ratio")) OR (tw:(survival)) OR (tw:("death toll")) OR (tw:("fatal outcome")) OR (tw:("acute respiratory infection")) OR (tw:(Patient Admi*)) OR (tw:(Voluntary Admission*)) OR (tw:(hospital Admi*)) OR (tw:(intensive care)) OR (tw:("Respiratory Care" Unit*)) OR (tw:(Recovery room*)) OR (tw:("close attention unit")) OR (tw:("critical care unit")) OR (tw:("intensive therapy unit")) OR (tw:("intensive treatment unit")) OR (tw:("special care unit")) OR (tw:(Respiration Artificial)) OR (tw:(Artificial Respirat*)) OR (tw:(Ventilation Mechanical)) OR (tw:(mechanical ventilation*)) OR (tw:("Interactive Ventilatory")) OR (tw:("Ventilatory Support")) OR (tw:("Ventilatory Assist")) OR (tw:("Assist Ventilation")) OR (tw:("Ventilation Proportional Assist")) OR (tw:("invasive ventilation")) OR (tw:("controlled respiration")) OR (tw:("controlled ventilation")) OR (tw:("mechanical respiration")) OR (tw:("mechanical ventilation")) OR (tw:(Inpatient*)) OR (tw:(in-patient*)) OR

(tw:(Hospitalised patient*)) OR (tw:(hospitalized patient*)) OR (tw:(in-hospital patient*)) OR (tw:(Risk Adjustment*)) OR (tw:("risk analysis")) OR (tw:("risk evaluation")) OR (tw:("safety assessment")) OR (tw:("Case-Mix" Adjustment*)) OR (tw:("Case Mix" Adjustment*)) OR (tw:(Outcomes Assessment*)) OR (tw:(Outcome Assessment*)) OR (tw:("Outcomes Research")) OR (tw:(Outcome Stud*)) OR (tw:(Outcome Measure*)) OR (tw:(severity)) OR (tw:("case-hospitalization ratio")) OR (tw:("proportion of hospitalization"))) AND la:("en") AND year_cluster:("2021" OR "2020") AND NOT db:("MEDLINE" OR "EMBASE") AND NOT type:("preprint") AND NOT ((clinical AND trial) OR mj:("Randomized Controlled Trials as Topic") OR type_of_study:("clinical_trials") OR random* OR randomization OR "therapeutic use") AND NOT ((case AND report*) OR type_of_study:("case_reports") OR "report a case" OR (report* AND ("outpatient department" OR (ambulatory AND care AND facilities) OR "ambulatory care facilities" OR clinic OR "clinic s" OR clinical OR clinically OR clinicals OR clinics OR patient*)) OR "reported case" OR (clinical AND presentation*) OR "patient management" OR (infected AND patient*)) AND NOT ((tw:("Psychological Outcomes")) OR (tw:("mental health")) OR (tw:("prevalence of depression")) OR (tw:("Psychosocial Impact")) OR (tw:(Depressi*)) OR (tw:(Melancholia*)) OR (tw:(Anxiety)) OR (tw:(Angst)) OR (tw:(Nervousness)) OR (tw:(Hypervigilance)) OR (tw:(Anxiousness)) OR (tw:(Anxieties)) OR mj:("Mental Health" OR "Depression" OR "Depressive Disorder"OR "Anxiety" OR "Anxiety Disorders")
