# Supplementary material for: Identification of methodological issues regarding direct impact indicators of COVID-19: a rapid scoping review on morbidity, severity and mortality
Source: Eur J Public Health. 2024 Jul 1;34(Suppl 1):i3–i10. doi: 10.1093/eurpub/ckae072 (PMC11215319; doi:10.1093/eurpub/ckae072)
Supplement: ckae072_Supplementary_Data [file ckae072_supplementary_data.zip › ejph-2023-06-phis-0310-File013.pdf]

Date of completion	Respondent's name	Respondent's surname	Respondent's country	Document title	Link to the document	Aim of the document (policy monitoring or decision tool document)	What type of indicators can you identify in your document? (multiple choice)	Type of morbidity indicators (multiple choice)	Type of severity indicators (multiple choice)	Type of mortality indicators (multiple choice)
10/19/2022 14:00:32	Amparo	Larrauri	Spain	Indicadores para la valoración de riesgo y niveles de Alerta de transmisión de COVID-19	https://www.sanidad.gob.es/profesionales/saludPublica/ccayes/alertasActual/nCov/documentos/Indicadores_de_riesgo_COVID.pdf	Prevention	Morbidity, Severity, Mortality	New cases in the population (e.g. incidence rate, cumulative incidence or COVID-19 case rate per million), Positivity rate (e.g. seroprevalence, active rate or % confirmed cases)	ICU (e.g. % Intensive care support, ICU admission rate or ICU admission)	Fatality rate (e.g. in-hospital mortality, percentage of deaths or case fatality ratio), Mortality rate (e.g. death from COVID-19, mortality within 48 hours or death rate)
10/28/2022 7:24:12	Hanna	Tolonen	Finland	Situation update on coronavirus	https://thl.fi/en/web/infectious-diseases-and-vaccinations/what-s-new/coronavirus-covid-19-latest-updates/situation-update-on-coronavirus	Promotion, Prevention, Care of COVID-19 patients	Morbidity, Severity, Mortality	New cases in the population (e.g. incidence rate, cumulative incidence or COVID-19 case rate per million), Positivity rate (e.g. seroprevalence, active rate or % confirmed cases)	ICU (e.g. % Intensive care support, ICU admission rate or ICU admission)	Mortality rate (e.g. death from COVID-19, mortality within 48 hours or death rate)
10/29/2022 15:42:24	Seila	Cilović Lagarija	Bosnia Herzegovina	we do not have official document like this, but have published report, according to some of these documents.	https://www.zzjzfbih.ba/wp-content/uploads/2022/10/Epidemiolo%C5%A1ki-izvje%C5%A1taj-24.10.2022..pdf	Prevention, Care of COVID-19 patients	Morbidity, Mortality, Composite (indicators mixing the above categories)	New cases in the population (e.g. incidence rate, cumulative incidence or COVID-19 case rate per million)	Ventilation procedures (e.g. mechanical ventilation, supplemental oxygen or ECMO rate), ICU (e.g. % Intensive care support, ICU admission rate or ICU admission)	Fatality rate (e.g. in-hospital mortality, percentage of deaths or case fatality ratio)

Date of completion	Respondent's name	Respondent's surname	Respondent's country	Document title	Link to the document	Aim of the document (policy monitoring or decision tool document)	What type of indicators can you identify in your document? (multiple choice)	Type of morbidity indicators (multiple choice)	Type of severity indicators (multiple choice)	Type of mortality indicators (multiple choice)
11/3/2022 15:44:42	Bachner	Florian	Austria	Corona-Ampel	https://corona-ampel.gv.at/	Prevention, Care of COVID-19 patients	Morbidity, Severity, Mortality, Composite (indicators mixing the above categories)	New cases in the population (e.g. incidence rate, cumulative incidence or COVID-19 case rate per million), Positivity rate (e.g. seroprevalence, active rate or % confirmed cases), New and pre-existing cases divided by population (e.g. case prevalence, seroprevalence or point prevalence of cases)	ICU (e.g. % Intensive care support, ICU admission rate or ICU admission), Length of stay (e.g. median length of stay, median duration of hospitalisation or ICU LOS)	Fatality rate (e.g. in-hospital mortality, percentage of deaths or case fatality ratio), Mortality rate (e.g. death from COVID-19, mortality within 48 hours or death rate)
11/3/2022 16:25:27	Martin	Thissen	Germany	Wöchentlicher Lagebericht des RKI zur Coronavirus-Krankheit-2019 (COVID-19)	https://www.rki.de/DE/Content/InfAZ/N/Neuartiges_Coronavirus/Situationsberichte/Wochenbericht/Wochenbericht_2022-10-27.pdf?__blob=publicationFile	Promotion, Prevention, Care of COVID-19 patients	Morbidity, Severity, Mortality	New cases in the population (e.g. incidence rate, cumulative incidence or COVID-19 case rate per million), Positivity rate (e.g. seroprevalence, active rate or % confirmed cases)	ICU (e.g. % Intensive care support, ICU admission rate or ICU admission)	Mortality rate (e.g. death from COVID-19, mortality within 48 hours or death rate)

Date of completion	Respondent's name	Respondent's surname	Respondent's country	Document title	Link to the document	Aim of the document (policy monitoring or decision tool document)	What type of indicators can you identify in your document? (multiple choice)	Type of morbidity indicators (multiple choice)	Type of severity indicators (multiple choice)	Type of mortality indicators (multiple choice)
11/3/2022 17:19:45	Luigi	Palmieri	Italy	IMPATTO DELL'EPIDEMIA COVID-19 SULLA MORTALITÀ TOTALE DELLA POPOLAZIONE RESIDENTE, PERIODO GENNAIO-NOVEMBRE 2020 (IMPACT OF THE COVID-19 EPIDEMIC ON THE TOTAL MORTALITY OF THE RESIDENT POPULATION PERIOD JANUARY-NOVEMBER 2020)	https://www.epicentro.iss.it/coronavirus/pdf/Rapp_Istat_Iss_gennaio-novembre-2020.pdf	Prevention	Morbidity, Mortality, Composite (indicators mixing the above categories)	New cases in the population (e.g. incidence rate, cumulative incidence or COVID-19 case rate per million), Positivity rate (e.g. seroprevalence, active rate or % confirmed cases), New and pre-existing cases divided by population (e.g. case prevalence, seroprevalence or point prevalence of cases)		Fatality rate (e.g. in-hospital mortality, percentage of deaths or case fatality ratio), Mortality rate (e.g. death from COVID-19, mortality within 48 hours or death rate)
11/3/2022 17:25:16	Luigi	Palmieri	Italy	EPIDEMIA COVID-19 Aggiornamento nazionale (appendice): 26/10/2022	https://www.epicentro.iss.it/coronavirus/bollettino/Bollettino-sorveglianza-integrata-COVID-19_26-ottobre-2022_appendix.pdf	Prevention	Morbidity	New cases in the population (e.g. incidence rate, cumulative incidence or COVID-19 case rate per million), New and pre-existing cases divided by population (e.g. case prevalence, seroprevalence or point prevalence of cases)		

Date of completion	Respondent's name	Respondent's surname	Respondent's country	Document title	Link to the document	Aim of the document (policy monitoring or decision tool document)	What type of indicators can you identify in your document? (multiple choice)	Type of morbidity indicators (multiple choice)	Type of severity indicators (multiple choice)	Type of mortality indicators (multiple choice)
11/3/2022 17:33:42	Luigi	Palmieri	Italy	REPORT ESTESO ISS COVID-19: SORVEGLIANZA, IMPATTO DELLE INFEZIONI ED EFFICACIA VACCINALE, Aggiornamento nazionale (EXTENDED ISS COVID-19 REPORT: SURVEILLANCE, IMPACT OF INFECTIONS AND VACCINAL EFFECTIVENESS, National update)	https://www.epicentro.iss.it/coronavirus/bollettino/Bollettino-sorveglianza-integrata-COVID-19_26-ottobre-2022.pdf	Prevention, Care of COVID-19 patients	Morbidity, Mortality	New cases in the population (e.g. incidence rate, cumulative incidence or COVID-19 case rate per million)		Fatality rate (e.g. in-hospital mortality, percentage of deaths or case fatality ratio), Mortality rate (e.g. death from COVID-19, mortality within 48 hours or death rate)
11/3/2022 17:43:08	Luigi	Palmieri	Italy	Characteristics of SARS-CoV-2 patients dying in Italy Report based on available data on January 10th, 2022	https://www.epicentro.iss.it/en/coronavirus/bollettino/Report-COVID-2019_10_january_2022.pdf	Prevention	Severity, Mortality		ICU (e.g. % Intensive care support, ICU admission rate or ICU admission), Clinical outcomes/ Complications (e.g. venous thromboembolism, ischemic stroke or % subjects with ARDS)	Mortality rate (e.g. death from COVID-19, mortality within 48 hours or death rate)

Date of completion	Respondent's name	Respondent's surname	Respondent's country	Document title	Link to the document	Aim of the document (policy monitoring or decision tool document)	What type of indicators can you identify in your document? (multiple choice)	Type of morbidity indicators (multiple choice)	Type of severity indicators (multiple choice)	Type of mortality indicators (multiple choice)
11/3/2022 18:06:37	Luigi	Palmieri	Italy	PRIMI RISULTATI DELL'INDAGINE DI SIEROPREVALENZA SUL SARS-CoV-2 (FIRST RESULTS OF THE SERIOUS PREVALENCE SURVEY ABOUT SARS-CoV-2)	https://www.istat.it/it/files/2020/08/ReportPrimiRisultatiIndagineSiero.pdf	Prevention	Morbidity	Positivity rate (e.g. seroprevalence, active rate or % confirmed cases), New and pre-existing cases divided by population (e.g. case prevalence, seroprevalence or point prevalence of cases)		
11/4/2022 9:28:02	Janis	Misins	Latvia	Ziņojums par Covid-19 izplatības risku novērtējumu un uz tiem balstītu lēmumu pieņemšanu par piesardzības un drošības pasākumu ieviešanu (Report on the assessment of the risks of the spread of Covid-19 and the adoption of decisions based on them on the implementation of precautionary and safety measures)	https://tap.mk.gov.lv/doc/2021_02/VMinf_030221_kriteriji_riski_.228.docx and https://www.vm.gov.lv/lv/jaunums/covid-19-drosibas-pasakumus-mazinas-saskana-ar-luksofora-principu	Promotion, Prevention, Care of COVID-19 patients	Morbidity, Composite (indicators mixing the above categories)	New cases in the population (e.g. incidence rate, cumulative incidence or COVID-19 case rate per million)		

Date of completion	Respondent's name	Respondent's surname	Respondent's country	Document title	Link to the document	Aim of the document (policy monitoring or decision tool document)	What type of indicators can you identify in your document? (multiple choice)	Type of morbidity indicators (multiple choice)	Type of severity indicators (multiple choice)	Type of mortality indicators (multiple choice)
11/4/2022 9:37:21	Claudia	Habl	Austria	Variantenmanagementplan (in English: Action Plan to tackle potential COVID-19 waves)	https://www.sozialministerium.at/dam/jcr:a56f7052-a3ab-4796-93f7-d0bae1eb92bf/220901_Variantenmanagementplan_pdfUA.pdf	Care of COVID-19 patients	Composite (indicators mixing the above categories)	New cases in the population (e.g. incidence rate, cumulative incidence or COVID-19 case rate per million), Positivity rate (e.g. seroprevalence, active rate or % confirmed cases), New and pre-existing cases divided by population (e.g. case prevalence, seroprevalence or point prevalence of cases)	ICU (e.g. % Intensive care support, ICU admission rate or ICU admission)	Mortality rate (e.g. death from COVID-19, mortality within 48 hours or death rate)
11/4/2022 10:21:28	Guy	Weber	Luxembourg	COVID-19: Rapports hebdomadaires	https://data.public.lu/fr/datasets/covid-19-rapports-hebdomadaires/	Promotion, Prevention, Care of COVID-19 patients	Morbidity, Severity, Mortality, Composite (indicators mixing the above categories)	New cases in the population (e.g. incidence rate, cumulative incidence or COVID-19 case rate per million), Positivity rate (e.g. seroprevalence, active rate or % confirmed cases), New and pre-existing cases divided by population (e.g. case prevalence, seroprevalence or point prevalence of cases)	ICU (e.g. % Intensive care support, ICU admission rate or ICU admission)	Fatality rate (e.g. in-hospital mortality, percentage of deaths or case fatality ratio), Mortality rate (e.g. death from COVID-19, mortality within 48 hours or death rate)

Date of completion	Respondent's name	Respondent's surname	Respondent's country	Document title	Link to the document	Aim of the document (policy monitoring or decision tool document)	What type of indicators can you identify in your document? (multiple choice)	Type of morbidity indicators (multiple choice)	Type of severity indicators (multiple choice)	Type of mortality indicators (multiple choice)
11/7/2022 11:35:08	SANDU	PETRU	Romania	N/A	https://insp.gov.ro/centrul-national-de-supraveghere-si-control-al-bolilor-transmisibile-cnscbt/	Promotion, Prevention, Care of COVID-19 patients	Morbidity, Mortality	New cases in the population (e.g. incidence rate, cumulative incidence or COVID-19 case rate per million), Positivity rate (e.g. seroprevalence, active rate or % confirmed cases), New and pre-existing cases divided by population (e.g. case prevalence, seroprevalence or point prevalence of cases)	Ventilation procedures (e.g. mechanical ventilation, supplemental oxygen or ECMO rate), ICU (e.g. % Intensive care support, ICU admission rate or ICU admission), Clinical outcomes/ Complications (e.g. venous thromboembolism, ischemic stroke or % subjects with ARDS), Length of stay (e.g. median length of stay, median duration of hospitalisation or ICU LOS)	Fatality rate (e.g. in-hospital mortality, percentage of deaths or case fatality ratio), Mortality rate (e.g. death from COVID-19, mortality within 48 hours or death rate)
11/7/2022 14:47:57	sarka	Dankova	Czech Republic	State of epidemy COVID-19: updated overview of trends	regular monitoring report (not official)	Prevention, Care of COVID-19 patients	Morbidity, Severity, Mortality, Composite (indicators mixing the above categories)	New cases in the population (e.g. incidence rate, cumulative incidence or COVID-19 case rate per million), Positivity rate (e.g. seroprevalence, active rate or % confirmed cases)	Ventilation procedures (e.g. mechanical ventilation, supplemental oxygen or ECMO rate), ICU (e.g. % Intensive care support, ICU admission rate or ICU admission)	Fatality rate (e.g. in-hospital mortality, percentage of deaths or case fatality ratio), Mortality rate (e.g. death from COVID-19, mortality within 48 hours or death rate)

Date of completion	Respondent's name	Respondent's surname	Respondent's country	Document title	Link to the document	Aim of the document (policy monitoring or decision tool document)	What type of indicators can you identify in your document? (multiple choice)	Type of morbidity indicators (multiple choice)	Type of severity indicators (multiple choice)	Type of mortality indicators (multiple choice)
11/7/2022 16:01:31	Pauline	White	Ireland	Resilience and Recovery 2020-2021: Plan for Living with COVID-19	https://www.gov.ie/en/publication/e5175-resilience-and-recovery-2020-2021-plan-for-living-with-covid-19/	Promotion, Prevention, Care of COVID-19 patients	Morbidity, Severity, Mortality	New cases in the population (e.g. incidence rate, cumulative incidence or COVID-19 case rate per million), Positivity rate (e.g. seroprevalence, active rate or % confirmed cases), New and pre-existing cases divided by population (e.g. case prevalence, seroprevalence or point prevalence of cases)	ICU (e.g. % Intensive care support, ICU admission rate or ICU admission)	Fatality rate (e.g. in-hospital mortality, percentage of deaths or case fatality ratio), Mortality rate (e.g. death from COVID-19, mortality within 48 hours or death rate)
11/8/2022 10:15:37	Jane	Idavain	Estonia	COVID-19 valmisolekukava 2022/2023 viirushooajaks (COVID-19 preparedness plan for the 2022/2023 virus season)	https://www.sm.ee/media/1873/download	Promotion, Prevention	Morbidity, Severity, Mortality, Composite (indicators mixing the above categories)	New cases in the population (e.g. incidence rate, cumulative incidence or COVID-19 case rate per million), New and pre-existing cases divided by population (e.g. case prevalence, seroprevalence or point prevalence of cases)	ICU (e.g. % Intensive care support, ICU admission rate or ICU admission), Length of stay (e.g. median length of stay, median duration of hospitalisation or ICU LOS)	Mortality rate (e.g. death from COVID-19, mortality within 48 hours or death rate)

Date of completion	Respondent's name	Respondent's surname	Respondent's country	Document title	Link to the document	Aim of the document (policy monitoring or decision tool document)	What type of indicators can you identify in your document? (multiple choice)	Type of morbidity indicators (multiple choice)	Type of severity indicators (multiple choice)	Type of mortality indicators (multiple choice)
11/8/2022 12:56:23	Jane	Idavain	Estonia	Koroonaviirus COVID-19 avaandmete kirjeldus (Description of open data on the coronavirus COVID-19)	https://www.terviseamet.ee/et/koroonaviirus/koroonaviiruse-andmestik		Morbidity, Severity, Mortality	New cases in the population (e.g. incidence rate, cumulative incidence or COVID-19 case rate per million), Positivity rate (e.g. seroprevalence, active rate or % confirmed cases), New and pre-existing cases divided by population (e.g. case prevalence, seroprevalence or point prevalence of cases)	Ventilation procedures (e.g. mechanical ventilation, supplemental oxygen or ECMO rate), ICU (e.g. % Intensive care support, ICU admission rate or ICU admission), Length of stay (e.g. median length of stay, median duration of hospitalisation or ICU LOS)	Mortality rate (e.g. death from COVID-19, mortality within 48 hours or death rate)
11/14/2022 18:11:16	Caroline	ALLEAUME	France	COVID-19 - Bilan 02 mars 2020 –07 novembre 2021	https://www.santepubliquefrance.fr/maladies-et-traumatismes/maladies-et-infections-respiratoires/infection-a-coronavirus/documents/bulletin-national/bulletin-de-sante-publique-covid-19.-juillet-2022	Promotion, Prevention, Care of COVID-19 patients	Morbidity, Mortality	New cases in the population (e.g. incidence rate, cumulative incidence or COVID-19 case rate per million), Positivity rate (e.g. seroprevalence, active rate or % confirmed cases), New and pre-existing cases divided by population (e.g. case prevalence, seroprevalence or point prevalence of cases)	ICU (e.g. % Intensive care support, ICU admission rate or ICU admission), Length of stay (e.g. median length of stay, median duration of hospitalisation or ICU LOS)	Mortality rate (e.g. death from COVID-19, mortality within 48 hours or death rate)

Date of completion	Respondent's name	Respondent's surname	Respondent's country	Document title	Link to the document	Aim of the document (policy monitoring or decision tool document)	What type of indicators can you identify in your document? (multiple choice)	Type of morbidity indicators (multiple choice)	Type of severity indicators (multiple choice)	Type of mortality indicators (multiple choice)
11/18/2022 13:00:37	Małgorzata	Strózyk-Kaczyńska	Poland	Infections and deaths in the context of vaccination (COVID-19)	https://basiw.mz.gov.pl/analizy/covid/statystyki-zakazen-i-zgonow-z-powodu-covid-19z-uwzglednieniem-zaszczepienia-przeciw-covid-19/	Morbidity, Mortality	New cases in the population (e.g. incidence rate, cumulative incidence or COVID-19 case rate per million)		Mortality rate (e.g. death from COVID-19, mortality within 48 hours or death rate)	Rate
11/18/2022 20:03:08	Jakov / Ivana	Vukovic / Pavic	Croatia	COVID-19 – izvješće HZJZ-a	https://www.hzjz.hr/aktualnosti/covid-19-izvjesce-hzjz-a/	Morbidity, Severity, Mortality, Composite (indicators mixing the above categories)	New cases in the population (e.g. incidence rate, cumulative incidence or COVID-19 case rate per million), Positivity rate (e.g. seroprevalence, active rate or % confirmed cases), New and pre-existing cases divided by population (e.g. case prevalence, seroprevalence or point prevalence of cases)	Ventilation procedures (e.g. mechanical ventilation, supplemental oxygen or ECMO rate), ICU (e.g. % Intensive care support, ICU admission rate or ICU admission)	Fatality rate (e.g. in-hospital mortality, percentage of deaths or case fatality ratio), Mortality rate (e.g. death from COVID-19, mortality within 48 hours or death rate)	Proportion, Rate, Count
11/21/2022 21:11:40	Luis	Lapao	Portugal	Relatório de Monitorização da Situação Epidemiológica da COVID-19	https://www.insa.min-saude.pt/relatorio-de-monitorizacao-da-situacao-epidemiologica-da-covid-19-17-08-2022/	Morbidity, Severity, Mortality	New cases in the population (e.g. incidence rate, cumulative incidence or COVID-19 case rate per million), Positivity rate (e.g. seroprevalence, active rate or % confirmed cases), New and pre-existing cases divided by population (e.g. case prevalence, seroprevalence or point prevalence of cases)	Ventilation procedures (e.g. mechanical ventilation, supplemental oxygen or ECMO rate), ICU (e.g. % Intensive care support, ICU admission rate or ICU admission)	Mortality rate (e.g. death from COVID-19, mortality within 48 hours or death rate)	Rate

Date of completion	Respondent's name	Respondent's surname	Respondent's country	Document title	Link to the document	Aim of the document (policy monitoring or decision tool document)	What type of indicators can you identify in your document? (multiple choice)	Type of morbidity indicators (multiple choice)	Type of severity indicators (multiple choice)	Type of mortality indicators (multiple choice)
11/22/2022 14:34:52	Lovisa	Syden	Sweden	Statistics on COVID-19 from NBHW and PHAS	https://www.socialstyrelsen.se/en/statistics-and-data/statistics/statistics-on-covid-19/ https://experience.arcgis.com/experience/09f821667ce64bf7be6f9f87457ed9aa	Morbidity, Severity, Mortality	Positivity rate (e.g. seroprevalence, active rate or % confirmed cases), New and pre-existing cases divided by population (e.g. case prevalence, seroprevalence or point prevalence of cases)	ICU (e.g. % Intensive care support, ICU admission rate or ICU admission), Length of stay (e.g. median length of stay, median duration of hospitalisation or ICU LOS)	Mortality rate (e.g. death from COVID-19, mortality within 48 hours or death rate)	Proportion, Rate, Count
11/22/2022 23:51:36	Gergely	Mikesy	Hungary	Information page about the coronavirus	https://koronavirus.gov.hu/	Morbidity, Severity, Mortality, Composite (indicators mixing the above categories)	New cases in the population (e.g. incidence rate, cumulative incidence or COVID-19 case rate per million), New and pre-existing cases divided by population (e.g. case prevalence, seroprevalence or point prevalence of cases)	ICU (e.g. % Intensive care support, ICU admission rate or ICU admission)	Mortality rate (e.g. death from COVID-19, mortality within 48 hours or death rate)	Count
11/23/2022 17:57:31	Sarah	Aldridge	United Kingdom	Coronavirus (COVID-19) latest insights<br>A live roundup of the latest data and trends about the coronavirus (COVID-19) pandemic from the ONS and other sources.	https://www.ons.gov.uk/peoplepopulationandcommunity/healthandsocialcare/conditionsanddiseases/articles/coronaviruscovid19/latestinsights	Morbidity, Severity, Mortality, Composite (indicators mixing the above categories)	Positivity rate (e.g. seroprevalence, active rate or % confirmed cases)	ICU (e.g. % Intensive care support, ICU admission rate or ICU admission)	Fatality rate (e.g. in-hospital mortality, percentage of deaths or case fatality ratio), Mortality rate (e.g. death from COVID-19, mortality within 48 hours or death rate)	Proportion, Rate, Count
11/25/2022 10:34:54	Metka	Zaletel	Slovenia	Covid-19 pandemic in Slovenia - Results of a panel online survey on the impact of the pandemic on life (SI-PANDA)	https://www.nijz.si/en/publikacije/covid-19-pandemic-in-slovenia-results-a-panel-online-survey-on-impact-pandemic-on-life-8 (this is an example of 21 reports)	Morbidity, Composite (indicators mixing the above categories)	Positivity rate (e.g. seroprevalence, active rate or % confirmed cases)			Proportion

Date of completion	Respondent's name	Respondent's surname	Respondent's country	Document title	Link to the document	Aim of the document (policy monitoring or decision tool document)	What type of indicators can you identify in your document? (multiple choice)	Type of morbidity indicators (multiple choice)	Type of severity indicators (multiple choice)	Type of mortality indicators (multiple choice)
11/25/2022 10:38:12	Metka	Zaletel	Slovenia	The first wave of covid-19 pandemic trough the prism of health statistics (Slovenian only)	https://www.nijz.si/sl/publikacije/prvi-val-epidemije-covida-19-skozi-prizmo-zdravstvene-statistike	Morbidity, Severity, Mortality, Composite (indicators mixing the above categories)	New cases in the population (e.g. incidence rate, cumulative incidence or COVID-19 case rate per million), Positivity rate (e.g. seroprevalence, active rate or % confirmed cases)	Ventilation procedures (e.g. mechanical ventilation, supplemental oxygen or ECMO rate), ICU (e.g. % Intensive care support, ICU admission rate or ICU admission), Length of stay (e.g. median length of stay, median duration of hospitalisation or ICU LOS)	Fatality rate (e.g. in-hospital mortality, percentage of deaths or case fatality ratio), Mortality rate (e.g. death from COVID-19, mortality within 48 hours or death rate)	Proportion, Rate, Count
11/28/2022 16:34:19	Miriam	Saso	Belgium	COVID-19 WEEKLY EPIDEMIOLOGICAL BULLETIN (25 NOVEMBRE 2022)	https://covid-19.sciensano.be/sites/default/files/Covid19/RAG_Derni%C3%A8re%20mise%20%C3%A0%20jour%20%C3%A9pid%C3%A9miologique_FR.pdf	Prevention, Care of COVID-19 patients	Morbidity, Severity, Mortality	New cases in the population (e.g. incidence rate, cumulative incidence or COVID-19 case rate per million), Positivity rate (e.g. seroprevalence, active rate or % confirmed cases), New and pre-existing cases divided by population (e.g. case prevalence, seroprevalence or point prevalence of cases)		Mortality rate (e.g. death from COVID-19, mortality within 48 hours or death rate)

Date of completion	Respondent's name	Respondent's surname	Respondent's country	Document title	Link to the document	Aim of the document (policy monitoring or decision tool document)	What type of indicators can you identify in your document? (multiple choice)	Type of morbidity indicators (multiple choice)	Type of severity indicators (multiple choice)	Type of mortality indicators (multiple choice)
11/28/2022 16:46:39	Miriam	Saso	Belgium	Assessment of the epidemiological situation - 30/12/2020	https://covid-19.sciensano.be/sites/default/files/Covid19/20201230_RAG_Update%20%C3%A9pid%C3%A9miologique_FR.pdf	Prevention, Care of COVID-19 patients	Morbidity, Severity, Mortality	New cases in the population (e.g. incidence rate, cumulative incidence or COVID-19 case rate per million), Positivity rate (e.g. seroprevalence, active rate or % confirmed cases), New and pre-existing cases divided by population (e.g. case prevalence, seroprevalence or point prevalence of cases)		Mortality rate (e.g. death from COVID-19, mortality within 48 hours or death rate)

Date of completion	Respondent's name	Respondent's surname	Respondent's country	Document title	Link to the document	Aim of the document (policy monitoring or decision tool document)	What type of indicators can you identify in your document? (multiple choice)	Type of morbidity indicators (multiple choice)	Type of severity indicators (multiple choice)	Type of mortality indicators (multiple choice)
11/28/2022 17:00:22	Miriam	Saso	Belgium	THEMATIC REPORT: KEY POINTS OF MONITORING HOSPITALIZED PATIENTS WITH COVID-19 INFECTION CONFIRMED - RESULTS FROM MARCH 15 TO JUNE 14, 2020 INCLUSIVE	https://covid-19.sciensano.be/sites/default/files/Covid19/COVID-19_THEMATIC%20REPORT_COVID-19%20HOSPITALISED%20PATIENTS_FR.pdf	Prevention, Care of COVID-19 patients	Morbidity, Severity, Mortality	New cases in the population (e.g. incidence rate, cumulative incidence or COVID-19 case rate per million), Positivity rate (e.g. seroprevalence, active rate or % confirmed cases), New and pre-existing cases divided by population (e.g. case prevalence, seroprevalence or point prevalence of cases)	Ventilation procedures (e.g. mechanical ventilation, supplemental oxygen or ECMO rate), ICU (e.g. % Intensive care support, ICU admission rate or ICU admission), Clinical outcomes/ Complications (e.g. venous thromboembolism, ischemic stroke or % subjects with ARDS), Length of stay (e.g. median length of stay, median duration of hospitalisation or ICU LOS)	Fatality rate (e.g. in-hospital mortality, percentage of deaths or case fatality ratio), Mortality rate (e.g. death from COVID-19, mortality within 48 hours or death rate)
11/28/2022 16:46:31	Tone	Bruun	Norway	Weekly reports (the most relevant document that are publicly available at present)	https://www.fhi.no/publ/2020/koronavirus-ukerapporter/	Prevention	Morbidity, Severity, Mortality, Composite (indicators mixing the above categories)	New cases in the population (e.g. incidence rate, cumulative incidence or COVID-19 case rate per million), Positivity rate (e.g. seroprevalence, active rate or % confirmed cases)	ICU (e.g. % Intensive care support, ICU admission rate or ICU admission)	
