# Supplementary material for: Identification of methodological issues regarding direct impact indicators of COVID-19: a rapid scoping review on morbidity, severity and mortality
Source: Eur J Public Health. 2024 Jul 1;34(Suppl 1):i3–i10. doi: 10.1093/eurpub/ckae072 (PMC11215319; doi:10.1093/eurpub/ckae072)
Supplement: ckae072_Supplementary_Data [file ckae072_supplementary_data.zip › ejph-2023-06-phis-0310-File005.pdf]

Supplementary material S1. Exclusion criteria

* None calculation is reported in the study nor papers including information collected from external

Exclusion criteria applied during the screening phase.

1. Not original research nor grey literature (i.e. editorials, protocols, or no original results)
2. Unrelated topics (e.g. an indirect impact indicator)
3. Not population-based studies (representative individuals of the general population). However, nursing homes, homes for the aged and inpatients (hospitalised patients) were included.
4. Subpopulations (e.g. paediatric patients, patients having a condition without comparison with general population, pregnant women, healthcare workers, etc.) However, elderly were included.
5. Duplicates.
6. Prognostic studies (i.e. forecasting studies, predictive models, prospective studies, projections and predictions, foresight, future)
7. Conference abstracts.

Exclusion criteria applied during the full-text reading phase.

1. Not population-based studies.
2. Studies not considering health indicators.
3. Studies not containing information on health indicators' calculation*.
4. Studies not having information of data sources used to get data for calculation.
5. Unrelated topics (e.g. an indirect impact indicator)
6. Subpopulations (e.g. paediatric patients, patients having a condition without comparison with general population, pregnant women, healthcare workers, students, US veterans, etc.) However, elderly were included.
7. Not original research nor grey literature nor conference abstract (i.e. editorial, protocol, or no original results)
8. Clinical trials or intervention studies.
9. Qualitative studies.
10. The study is a continuation of a previous study. Studies were selected among those providing more information regarding health indicators. If there were several studies related but using at least one different indicator, all those papers were kept.

Websites providing their own indicator's calculation. Some of the most often used websites were:

- https://www.worldometers.info/ (Worldometer Team)
- https://coronavirus.jhu.edu/map.html (John Hopkins University)
- https://ourworldindata.org/ (University of Oxford)
